# Supplementary material for: An autism spectrum disorder mutation in Topoisomerase 3β causes accumulation of covalent mRNA intermediates by disrupting metal binding within the zinc finger domain
Source: Nucleic Acids Res. 2025 Nov 4;53(20):gkaf1138. doi: 10.1093/nar/gkaf1138 (PMC12585913; doi:10.1093/nar/gkaf1138)
Supplement: gkaf1138_Supplemental_Files [file gkaf1138_supplemental_files.zip › resub Warrick et al (2025)_TOP3B C666R_Supplementary Data.docx]

**Supplementary Data for Warrick *et al*.**

**An autism spectrum disorder mutation in Topoisomerase 3β causes accumulation of covalent mRNA intermediates by disrupting metal binding within the zinc finger domain**

**Supplementary Figures**


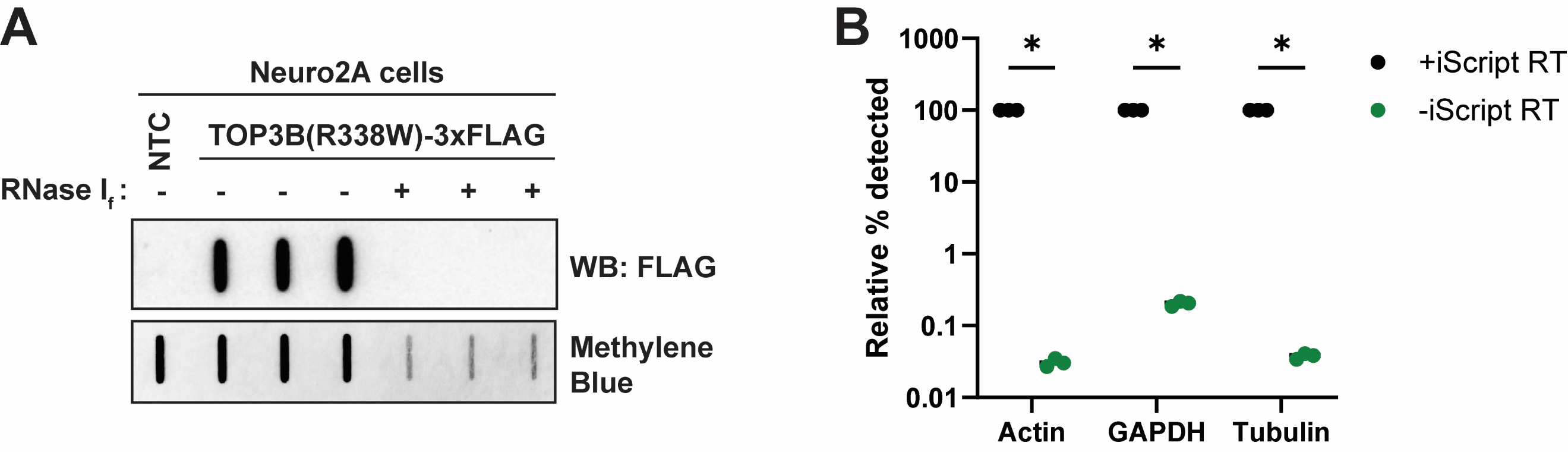


**Supplementary Figure S1. Cell-based TOP3B activity assay is highly sensitive to RNase and is not contaminated with DNA.** A) Anti-FLAG slot blot of TOP3B-R338W•mRNA covalent intermediates isolated from Neuro2A cells via oligo-dT magnetic beads under stringent conditions (**Figure 1C**) without and with addition of RNase I_f_ during the last wash (see methods for more details) (nitrocellulose membrane). Free mRNA was stained with methylene blue (positively charged nylon membrane) and served as a loading control. The decrease in methylene blue staining in the +RNase I_f_ lanes is expected due to the RNase activity during isolation; equal volume of eluates were assessed (see methods). B) Relative quantification by RT-qPCR of Actin, GAPDH, and Tubulin mRNA with and without iScript Reverse Transcriptase (RT) added during cDNA synthesis. 0.2 ng of mEGFP plasmid was added prior to cDNA synthesis and used as reference gene that was independent of RT. n = 3 biological replicates. Comparisons were made using an unpaired t test with Welch correction. * = p<0.000001. Exact p-values are reported in **Supplementary Table S9**.


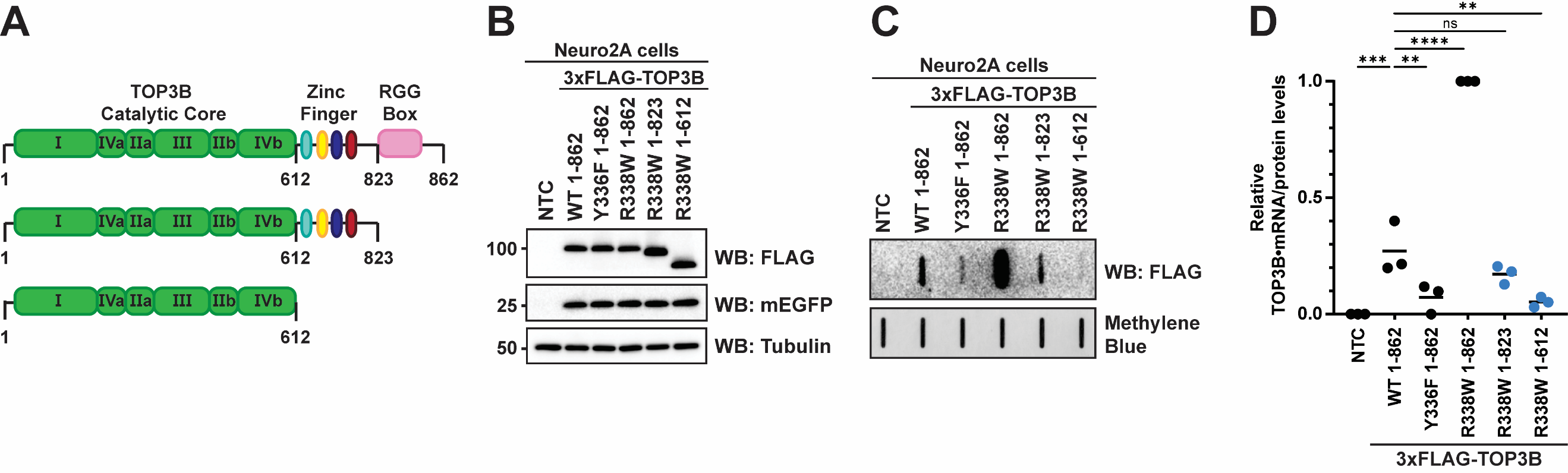


**Supplementary Figure S2. Deletion of C-terminal domains affects the accumulation of TOP3B-R338W•mRNA covalent intermediates.** A) Diagram of full-length and mutant TOP3B harboring domain deletions used. B) Anti-FLAG Western blot of WT and mutant 3xFLAG-TOP3B in Neuro2A cells. mEGFP was used as a transfection control and tubulin was used as a loading control. NTC = no template control. C) Anti-FLAG slot blot of WT and mutant TOP3B•mRNA covalent intermediates isolated from Neuro2A cells (nitrocellulose membrane). Free mRNA was stained with methylene blue (positively charged nylon membrane) and served as a loading control. D) TOP3B activity levels were assessed by quantifying TOP3B•mRNA covalent intermediate levels (signal in panel C) normalized by steady state protein levels (signal in panel B). 3xFLAG-TOP3B protein levels were first normalized by the mEGFP transfection control. Data were then set relative to the R338W mutant. n = 3 biological replicates. Comparisons were made using a one-way ANOVA with Dunnett’s multiple comparisons. ** = p<0.01, *** = p≤0.001, **** = p≤0.0001. ns = not significant. Exact p-values are reported in **Supplementary Table S9**.


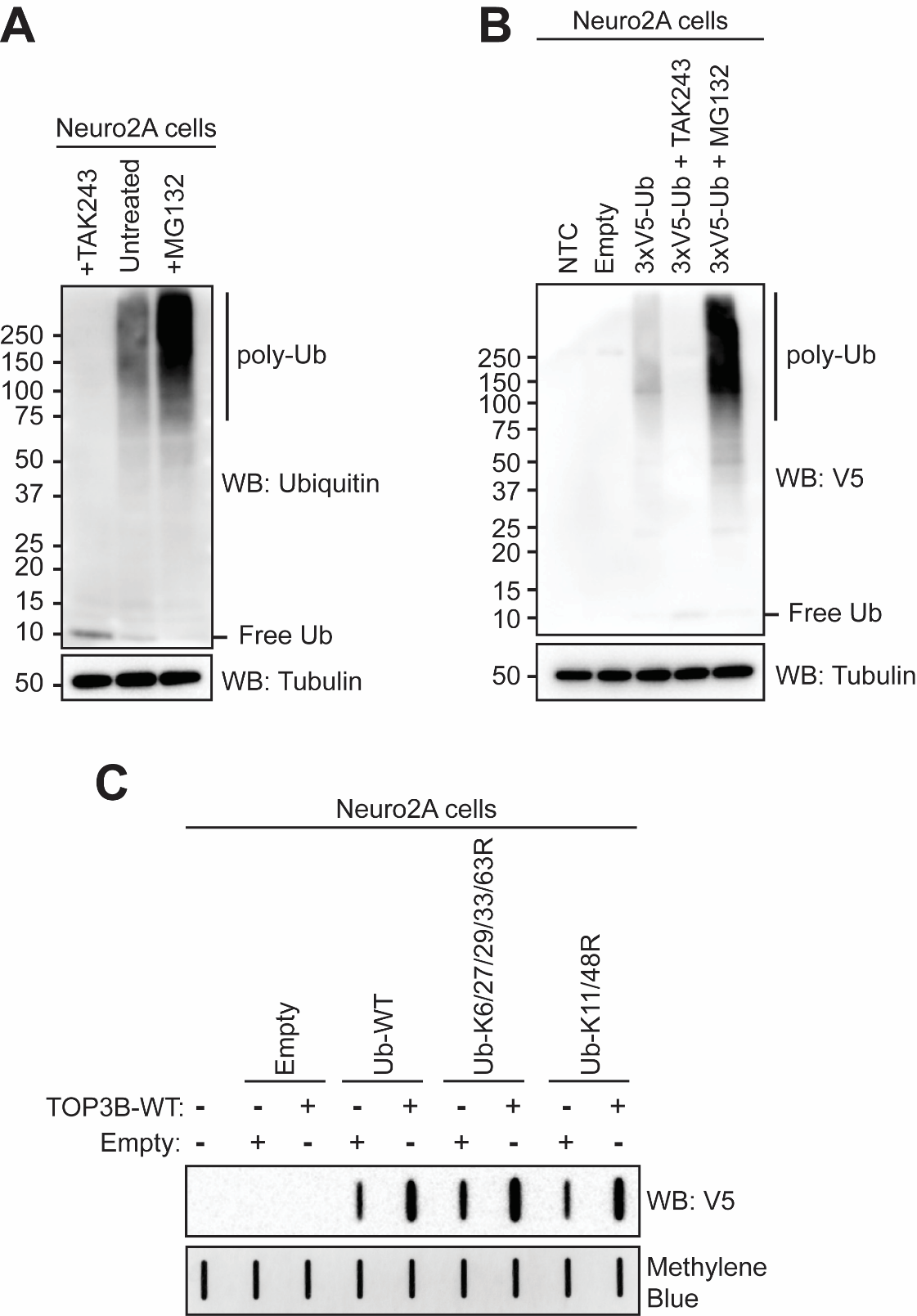


**Supplementary Figure S3. 3xV5-Ubiquitin is functional and incorporated into polyubiquitin chains.** A) Anti-Ubiquitin (Ub) Western blot of Neuro2A cells treated with 10 µM TAK243 or MG132 for 2 hrs. Tubulin was used as a loading control. B) Anti-V5 Western blot from Neuro2A cell lysates transfected with an empty plasmid or a plasmid encoding 3xV5-Ub for 24 hrs and then left untreated or treated with 10 µM TAK243 or MG132 for 2 hrs. Tubulin was used as a loading control. C) Anti-V5 slot blot detecting TOP3B•mRNA covalent intermediates isolated from Neuro2A cells transfected with an empty plasmid control (Empty) and or a plasmid encoding WT TOP3B-3xFLAG. An empty plasmid control (Empty) or a plasmid encoding WT or mutant 3xV5-Ubiquitin (3xV5-Ub) was co-expressed. Free mRNA was stained with methylene blue and served as a loading control.


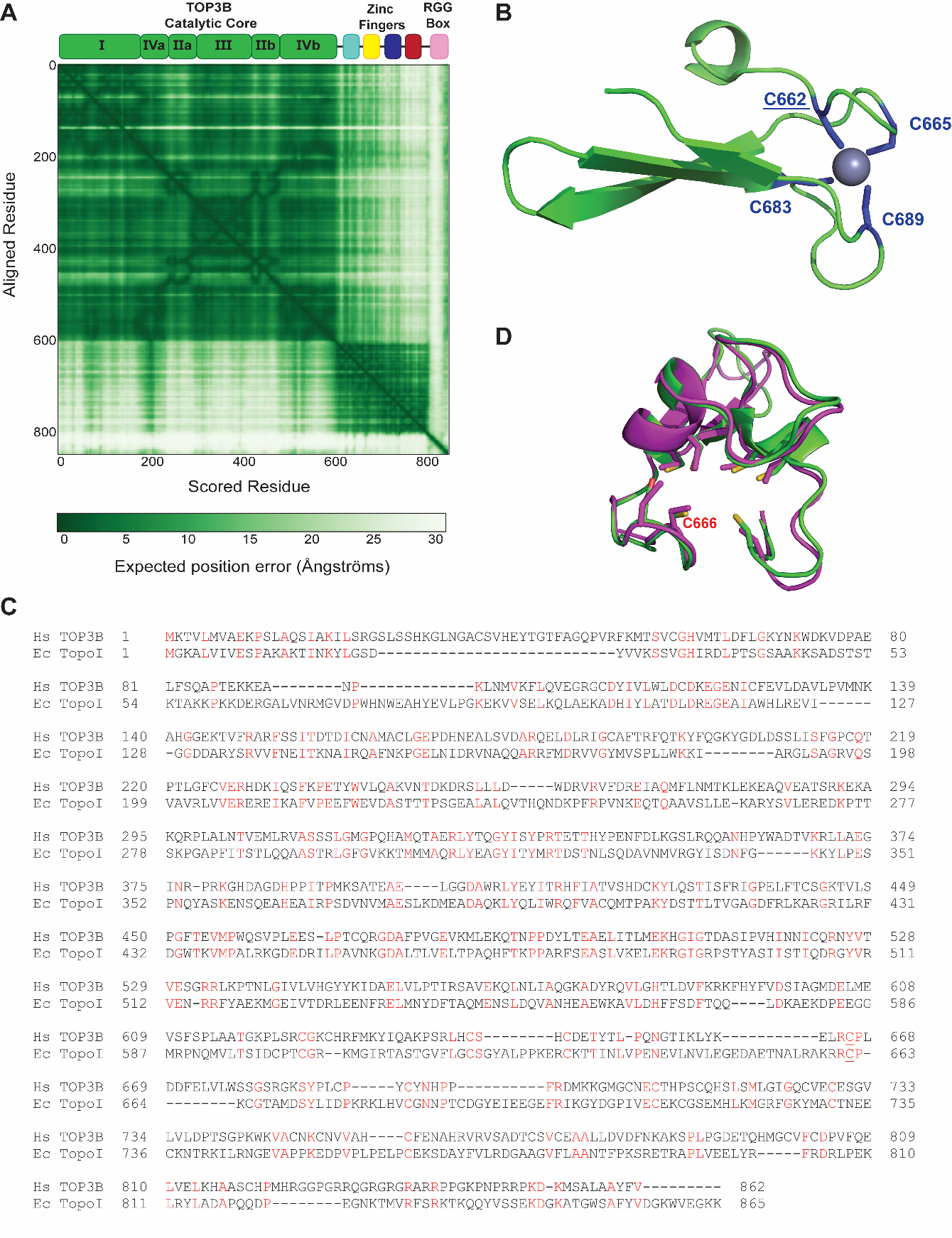


**Supplementary Figure S4. Assessment of the AlphaFold predicted structure of TOP3B and conservation of the C666 residue within a metal binding motif.** A) Predicted Alignment Error (PAE) plot for the predicted structure of TOP3B (UniProtKB: O95985) shows high accuracy (low error) for the TOP3B catalytic core region. Zinc fingers show low positional accuracy relative to each other but high optional error relative to the catalytic core, and the RGG motif shows low positional accuracy. B-C) The homologous C666 residue in human TOP3B is conserved in *E. coli* TopoI within a *bona fide* C4-type zinc binding motif. The solved X-ray crystallography structure of full-length *E. coli* TopoI (PDB: 4RUL) demonstrates that C662 (underlined) contributes to a C4-type zinc finger motif; for simplicity, only residues 655-696 are shown (B). Alignment of human TOP3B and *E. coli* TopoI using COBALT (Constraint-based Multiple Alignment Tool) from NCBI with sequence identity shown in red (C). The Human TOP3B (Hs TOP3B) sequence is from NCBI Reference Sequence NP_003926.1 and the *E. coli* TopoI (Ec TopoI) sequence is from NCBI Reference Sequence NP_003926.1. The C666 residue in human TOP3B and the homologous C662 residue in *E. coli* TopoI are underlined. D) Pymol structural alignment of the AlphaFold 3 prediction (green) and the published cryo-EM structure (magenta) for TOP3B (PDB: 9CAH); only residues 663-713 are shown for simplicity and to compare the D1C3 motif that consists of C666 (labeled in red as a reference point).


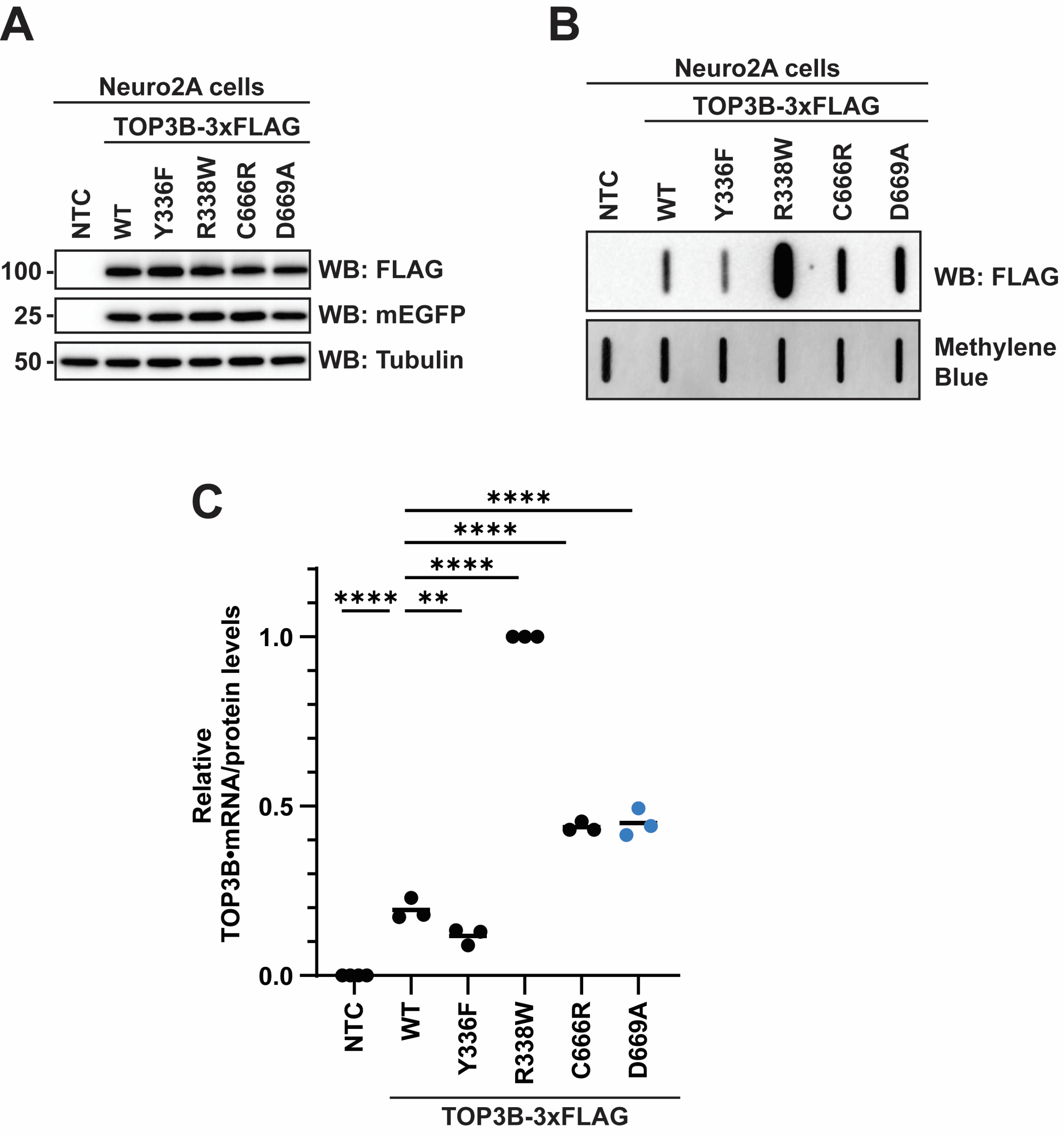


**Supplementary Figure S5. Mutating the D669 residue within the D1C3 motif phenocopies the C666R mutation.** A) Anti-FLAG Western blot of WT and mutant TOP3B-3xFLAG in Neuro2A cells. mEGFP was used as a transfection control and tubulin was used as a loading control. NTC = no template control. B) Anti-FLAG slot blot of WT and mutant TOP3B•mRNA covalent intermediates isolated from Neuro2A cells (nitrocellulose membrane). Free mRNA was stained with methylene blue (positively charged nylon membrane) and served as a loading control. C) TOP3B activity levels were assessed by quantifying TOP3B•mRNA covalent intermediate levels (signal in panel B) normalized by steady state protein levels (signal in panel A). TOP3B-3xFLAG protein levels were first normalized by the mEGFP transfection control. Data were then set relative to the R338W mutant. n = 3 biological replicates. Comparisons were made using a one-way ANOVA with Dunnett’s multiple comparisons. ** = p<0.01, **** = p≤0.0001. Exact p-values are reported in **Supplementary Table S9**.

**
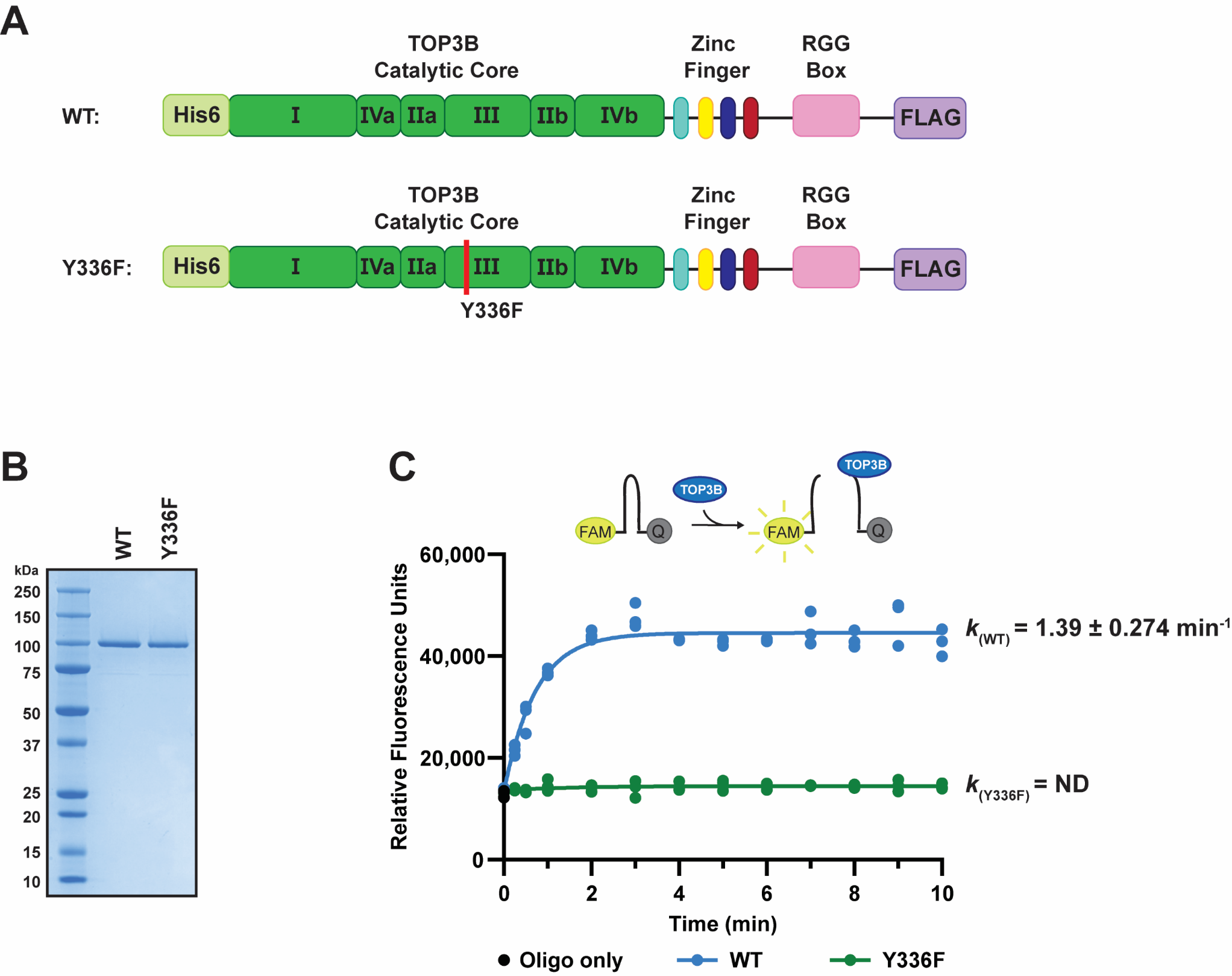
**

**Supplementary Figure S6. Recombinant full-length TOP3B is catalytically active.** A) Schematic of recombinant full-length His6-TOP3B(WT)-FLAG and His6-TOP3B(Y336F)-FLAG expressed and purified using a baculovirus-insect cell system. B) SDS-PAGE and Coomassie stain of the indicated recombinant full-length TOP3B. C) Diagram of quencher release assay demonstrating that catalytic cleavage of stem-loop DNA substrate by TOP3B separates the quencher and fluorophore to allow FAM fluorescence (top). Quencher release assay demonstrating recombinant TOP3B-WT is catalytically active (bottom). n = 3 biological replicates.


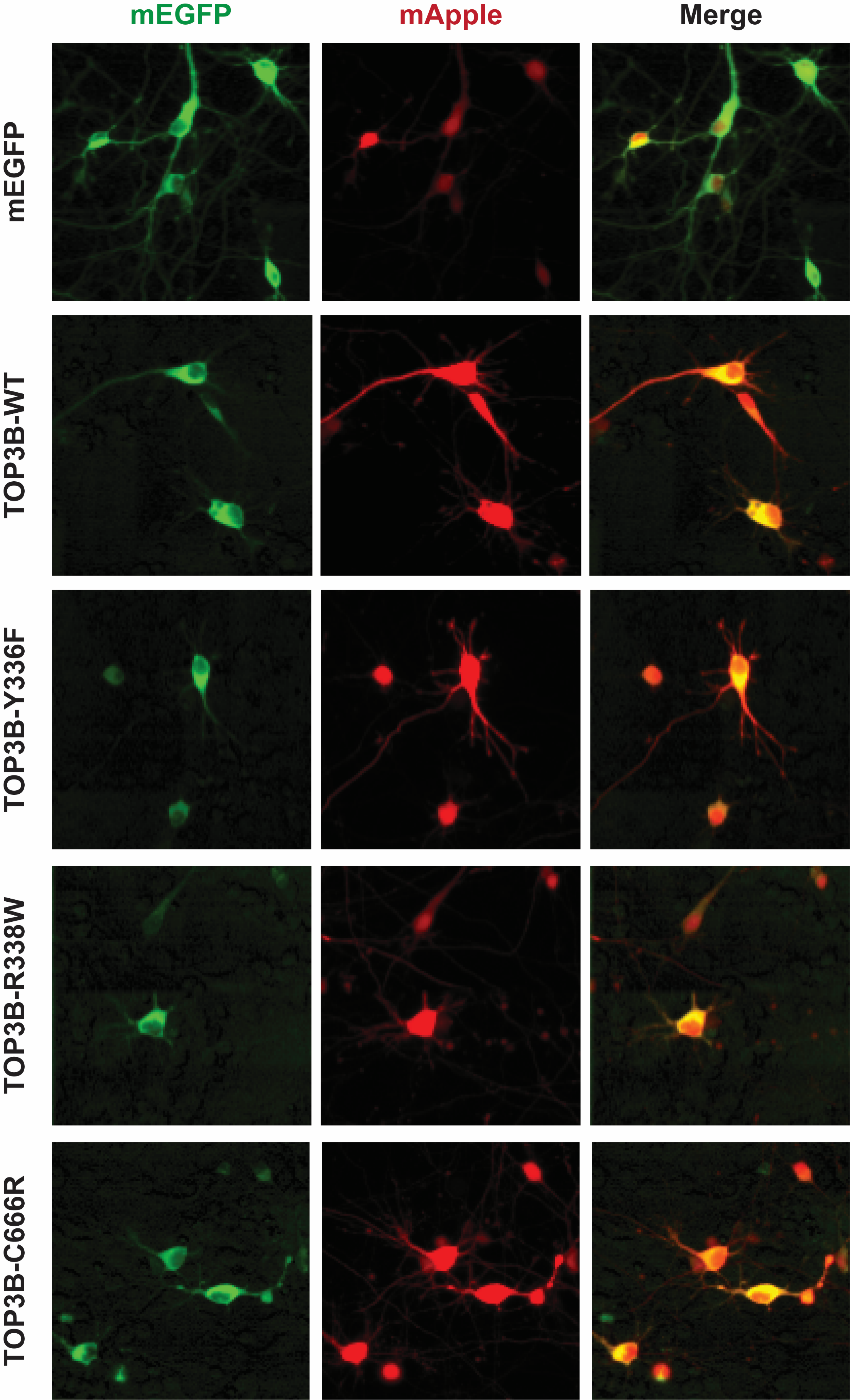


**Supplementary Figure S7.** **mEGFP-NES and mEGFP-NES-TOP3B fusion proteins are predominantly cytoplasmic in primary neurons.** Fluorescence microscopy of primary rat cortical neurons expressing mEGFP-NES and mEGFP-NES-TOP3B-WT, -Y336F, -R338W, and -C666R. mApple is co-transfected to mark the neuron body.


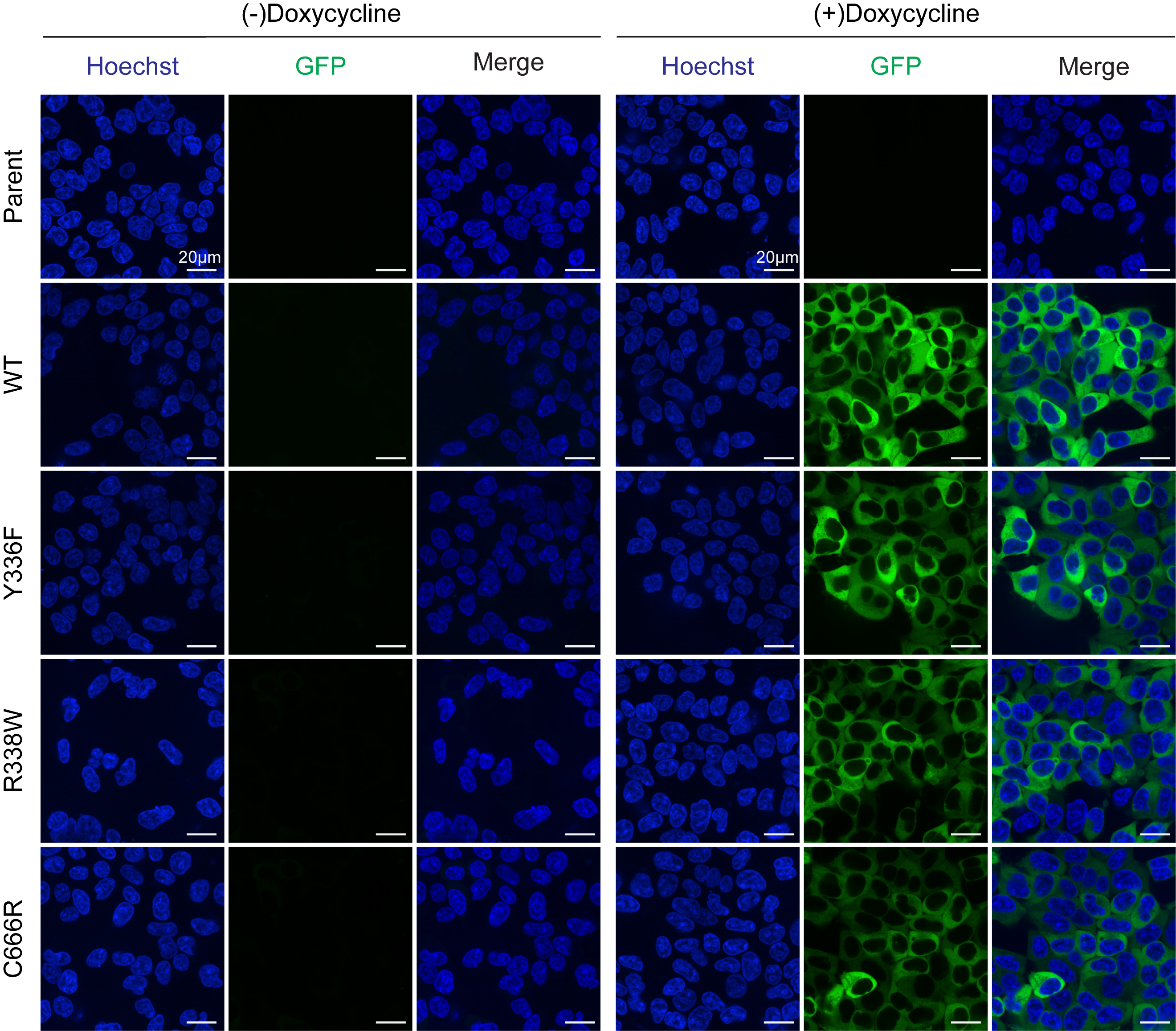


**Supplementary Figure S8. Doxycycline-inducible TOP3B is primarily cytoplasmic.** Spinning disk confocal microscopy of the parental Flp-In T-REx 293 cell line and stable cell lines with inducible expression of 3xFLAG-mEGFP-NES-TOP3B (WT, Y336F, and R338W, and C666R). Where indicated, expression was induced with doxycycline (Dox; 1 µg/mL final) for 48 hrs. DNA was stained using Hoechst 33342 (Hoechst; 1 µg/mL final). Images were taken at 60X magnification.


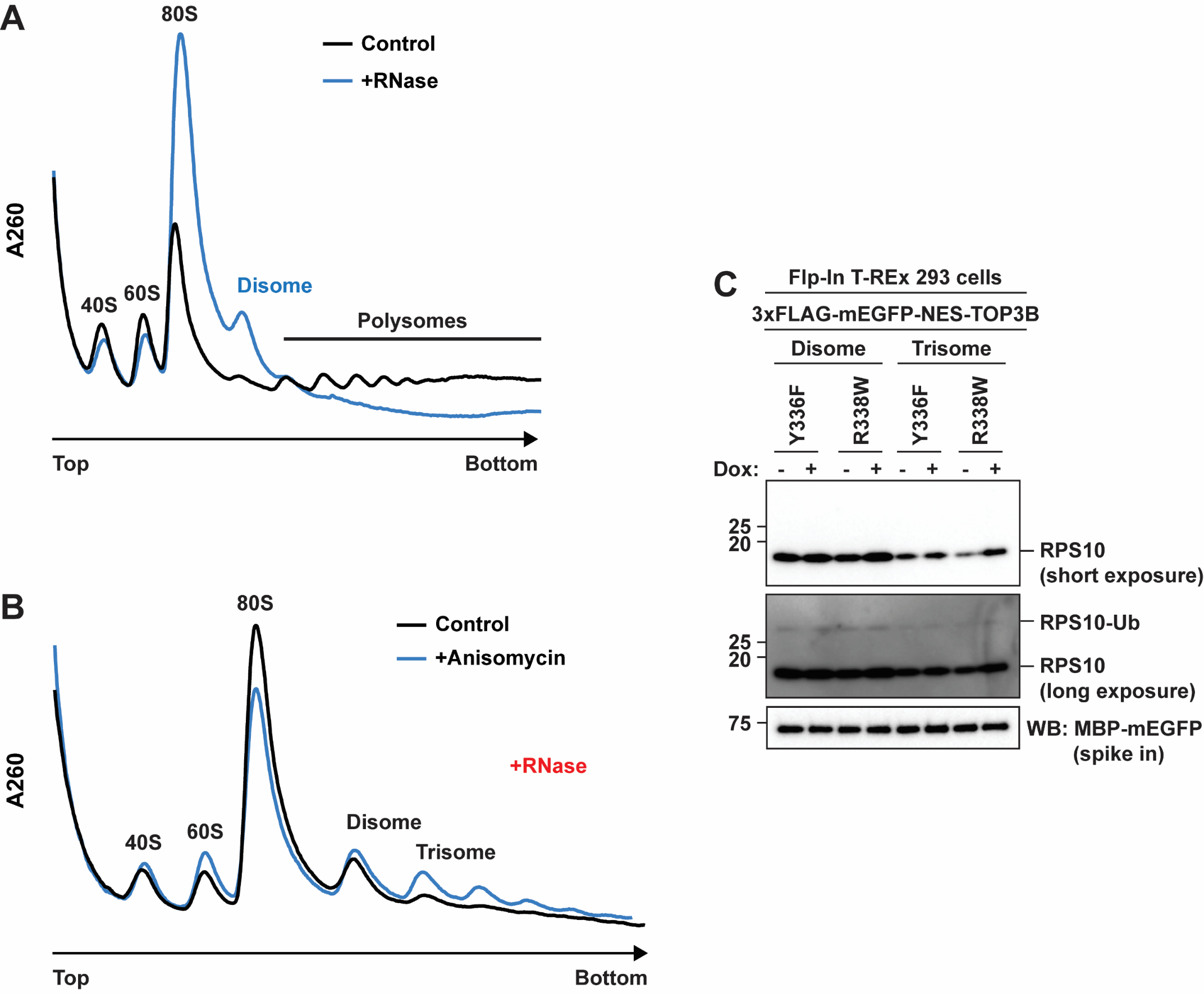


**Supplementary Figure S9. Micrococcal nuclease treatment effectively eliminates polysomes and allows for the detection of nuclease-resistant collied ribosomes.** A) Polysome analysis (10-50% (w/v) sucrose gradient) of uninduced 3xFLAG-mEGFP-NES-TOP3B (Y336F) Flp-In T-REx 293 cells. Lysates were either mock untreated (Control) or treated with S7 micrococcal nuclease (+RNase). B) Polysome analysis of uninduced 3xFLAG-mEGFP-NES-TOP3B (Y336F) Flp-In T-REx 293 cells treated with vehicle or intermediate dose of anisomycin (0.2 µg/mL final) for 15 min prior to harvesting. Both lysates were treated with S7 micrococcal nuclease for detection of nuclease-resistant ribosome collisions. C) Anti-RPS10 Western blot showing the expected ubiquitylation of RPS10 caused by collided ribosomes.

**OPEN reading frames used in this manuscript**

Full length TOP3B sequence

Point mutation

MYC tag

3xFLAG tag

mEGFP

V5 tag

Ubiquitin

NES

MBP

TEV

FLAG tag

Zinc finger only of TOP3B

Strep tag

His6 tag

**ORFs in plasmids for transfection of Neuro2A cells**

**TOP3B-3xFLAG WT**

ATGAAGACTGTGCTCATGGTTGCTGAAAAGCCGTCCTTGGCACAGTCAATTGCCAAAATCCTCTCTAGAGGGAGCCTGTCCTCACACAAAGGGCTGAACGGGGCCTGCTCAGTCCACGAGTACACTGGGACCTTTGCTGGCCAGCCAGTGCGCTTCAAGATGACGTCTGTCTGTGGTCACGTGATGACCCTGGATTTCCTGGGAAAATACAACAAATGGGACAAAGTGGACCCCGCAGAACTGTTCAGCCAAGCTCCCACGGAGAAGAAAGAAGCTAACCCCAAGCTGAACATGGTGAAGTTCCTGCAGGTGGAGGGCAGAGGCTGCGACTACATCGTGCTGTGGCTGGACTGCGACAAGGAGGGGGAGAACATCTGCTTTGAGGTTCTTGATGCTGTTCTGCCCGTCATGAACAAGGCCCATGGTGGCGAGAAGACCGTGTTCCGGGCCAGGTTTAGCTCCATCACGGACACAGACATCTGTAATGCCATGGCCTGCCTAGGCGAGCCTGACCACAACGAGGCGCTCTCAGTGGATGCTCGCCAGGAGCTGGACCTGCGAATCGGCTGTGCATTCACCAGGTTTCAGACTAAATATTTCCAGGGGAAATACGGTGATTTAGACAGCTCTCTCATCTCCTTTGGGCCGTGTCAGACTCCAACCCTGGGATTCTGTGTGGAGAGACATGATAAAATCCAGTCCTTCAAACCAGAGACCTACTGGGTGCTGCAGGCCAAGGTTAACACTGACAAAGACAGATCTCTCCTTTTGGACTGGGACCGAGTAAGAGTGTTTGACCGGGAGATCGCACAGATGTTTTTAAACATGACAAAGCTGGAGAAGGAAGCCCAGGTGGAGGCCACAAGCAGGAAAGAAAAGGCCAAGCAGAGGCCCCTGGCCCTGAACACTGTGGAGATGCTGCGTGTGGCCAGCTCTTCTCTGGGCATGGGGCCGCAGCACGCCATGCAGACGGCTGAGCGGCTCTACACGCAAGGCTACATCAGCTACCCACGGACAGAGACCACCCACTACCCTGAGAACTTTGACCTGAAGGGCTCTCTGCGGCAGCAGGCCAACCACCCCTACTGGGCCGACACGGTGAAGCGGTTGTTAGCAGAAGGTATCAACCGCCCGCGGAAAGGCCATGACGCCGGCGACCATCCCCCCATCACCCCCATGAAGTCTGCCACAGAGGCCGAATTAGGGGGTGACGCGTGGCGGCTCTATGAGTACATCACCAGACACTTCATCGCCACGGTCAGCCATGACTGCAAGTACCTGCAGAGCACCATCTCCTTCAGAATTGGGCCCGAGCTCTTCACCTGCTCCGGGAAGACCGTCCTCTCACCAGGCTTCACGGAGGTCATGCCCTGGCAGAGCGTGCCCCTGGAGGAGAGCCTGCCCACTTGCCAGCGGGGTGATGCCTTCCCTGTGGGCGAGGTGAAGATGCTGGAGAAGCAGACGAACCCACCCGACTACCTGACGGAGGCCGAGCTCATCACGCTCATGGAGAAGCATGGCATCGGCACGGATGCCAGCATCCCTGTGCATATCAACAACATCTGCCAGCGCAACTATGTCACGGTGGAGAGCGGGCGCCGGCTCAAGCCCACCAACCTCGGCATCGTCCTGGTGCACGGCTACTATAAGATTGATGCAGAGCTGGTGCTCCCCACCATCCGCAGTGCAGTGGAGAAGCAGCTGAACCTGATCGCCCAGGGCAAGGCCGACTACCGCCAGGTCCTGGGCCACACCCTGGACGTGTTCAAGAGGAAGTTCCACTACTTTGTCGACTCCATTGCTGGCATGGATGAGTTGATGGAGGTGTCTTTCTCGCCCCTGGCGGCCACAGGCAAGCCCCTCTCACGCTGTGGGAAGTGCCACCGCTTCATGAAGTACATCCAGGCCAAGCCAAGCCGCCTGCACTGCTCCCACTGCGATGAGACCTACACGCTCCCCCAGAACGGCACCATCAAGCTCTACAAGGAGCTCCGCTGCCCTCTGGATGACTTCGAGCTGGTCCTGTGGTCATCAGGCTCTCGGGGCAAGAGCTACCCGCTGTGCCCCTACTGCTACAACCACCCACCCTTCCGAGACATGAAGAAAGGCATGGGCTGCAACGAGTGTACGCACCCCTCCTGCCAGCACTCGCTGAGCATGCTGGGCATCGGCCAGTGCGTGGAATGTGAGAGCGGGGTGCTGGTGCTGGACCCCACCTCGGGCCCCAAGTGGAAGGTGGCCTGCAACAAGTGCAACGTGGTAGCGCACTGCTTCGAGAACGCCCACCGCGTGCGGGTGTCCGCCGACACCTGCAGTGTCTGTGAGGCCGCCTTGCTTGATGTGGACTTCAACAAGGCCAAGTCCCCACTCCCGGGCGATGAGACGCAGCACATGGGCTGCGTCTTTTGTGACCCCGTCTTCCAGGAGCTGGTGGAGCTGAAGCATGCGGCCTCCTGCCACCCCATGCACCGCGGTGGACCAGGGAGAAGGCAGGGTCGAGGGCGGGGCCGGGCCAGGAGGCCCCCTGGGAAGCCCAACCCCAGACGGCCCAAGGACAAGATGTCAGCCCTGGCCGCCTACTTTGTAAGCGGACCGACGCGTACGCGGCCGCTCGAGCAGAAACTCATCTCAGAAGAGGATCTGGCAGCAAATGATATCCTGgactacaaagaccatgacggtgattataaagatcatgacatcgattacaaggatgacgatgacaagGTTTAA

**TOP3B-3xFLAG Y336F**

ATGAAGACTGTGCTCATGGTTGCTGAAAAGCCGTCCTTGGCACAGTCAATTGCCAAAATCCTCTCTAGAGGGAGCCTGTCCTCACACAAAGGGCTGAACGGGGCCTGCTCAGTCCACGAGTACACTGGGACCTTTGCTGGCCAGCCAGTGCGCTTCAAGATGACGTCTGTCTGTGGTCACGTGATGACCCTGGATTTCCTGGGAAAATACAACAAATGGGACAAAGTGGACCCCGCAGAACTGTTCAGCCAAGCTCCCACGGAGAAGAAAGAAGCTAACCCCAAGCTGAACATGGTGAAGTTCCTGCAGGTGGAGGGCAGAGGCTGCGACTACATCGTGCTGTGGCTGGACTGCGACAAGGAGGGGGAGAACATCTGCTTTGAGGTTCTTGATGCTGTTCTGCCCGTCATGAACAAGGCCCATGGTGGCGAGAAGACCGTGTTCCGGGCCAGGTTTAGCTCCATCACGGACACAGACATCTGTAATGCCATGGCCTGCCTAGGCGAGCCTGACCACAACGAGGCGCTCTCAGTGGATGCTCGCCAGGAGCTGGACCTGCGAATCGGCTGTGCATTCACCAGGTTTCAGACTAAATATTTCCAGGGGAAATACGGTGATTTAGACAGCTCTCTCATCTCCTTTGGGCCGTGTCAGACTCCAACCCTGGGATTCTGTGTGGAGAGACATGATAAAATCCAGTCCTTCAAACCAGAGACCTACTGGGTGCTGCAGGCCAAGGTTAACACTGACAAAGACAGATCTCTCCTTTTGGACTGGGACCGAGTAAGAGTGTTTGACCGGGAGATCGCACAGATGTTTTTAAACATGACAAAGCTGGAGAAGGAAGCCCAGGTGGAGGCCACAAGCAGGAAAGAAAAGGCCAAGCAGAGGCCCCTGGCCCTGAACACTGTGGAGATGCTGCGTGTGGCCAGCTCTTCTCTGGGCATGGGGCCGCAGCACGCCATGCAGACGGCTGAGCGGCTCTACACGCAAGGCTACATCAGCtttCCACGGACAGAGACCACCCACTACCCTGAGAACTTTGACCTGAAGGGCTCTCTGCGGCAGCAGGCCAACCACCCCTACTGGGCCGACACGGTGAAGCGGTTGTTAGCAGAAGGTATCAACCGCCCGCGGAAAGGCCATGACGCCGGCGACCATCCCCCCATCACCCCCATGAAGTCTGCCACAGAGGCCGAATTAGGGGGTGACGCGTGGCGGCTCTATGAGTACATCACCAGACACTTCATCGCCACGGTCAGCCATGACTGCAAGTACCTGCAGAGCACCATCTCCTTCAGAATTGGGCCCGAGCTCTTCACCTGCTCCGGGAAGACCGTCCTCTCACCAGGCTTCACGGAGGTCATGCCCTGGCAGAGCGTGCCCCTGGAGGAGAGCCTGCCCACTTGCCAGCGGGGTGATGCCTTCCCTGTGGGCGAGGTGAAGATGCTGGAGAAGCAGACGAACCCACCCGACTACCTGACGGAGGCCGAGCTCATCACGCTCATGGAGAAGCATGGCATCGGCACGGATGCCAGCATCCCTGTGCATATCAACAACATCTGCCAGCGCAACTATGTCACGGTGGAGAGCGGGCGCCGGCTCAAGCCCACCAACCTCGGCATCGTCCTGGTGCACGGCTACTATAAGATTGATGCAGAGCTGGTGCTCCCCACCATCCGCAGTGCAGTGGAGAAGCAGCTGAACCTGATCGCCCAGGGCAAGGCCGACTACCGCCAGGTCCTGGGCCACACCCTGGACGTGTTCAAGAGGAAGTTCCACTACTTTGTCGACTCCATTGCTGGCATGGATGAGTTGATGGAGGTGTCTTTCTCGCCCCTGGCGGCCACAGGCAAGCCCCTCTCACGCTGTGGGAAGTGCCACCGCTTCATGAAGTACATCCAGGCCAAGCCAAGCCGCCTGCACTGCTCCCACTGCGATGAGACCTACACGCTCCCCCAGAACGGCACCATCAAGCTCTACAAGGAGCTCCGCTGCCCTCTGGATGACTTCGAGCTGGTCCTGTGGTCATCAGGCTCTCGGGGCAAGAGCTACCCGCTGTGCCCCTACTGCTACAACCACCCACCCTTCCGAGACATGAAGAAAGGCATGGGCTGCAACGAGTGTACGCACCCCTCCTGCCAGCACTCGCTGAGCATGCTGGGCATCGGCCAGTGCGTGGAATGTGAGAGCGGGGTGCTGGTGCTGGACCCCACCTCGGGCCCCAAGTGGAAGGTGGCCTGCAACAAGTGCAACGTGGTAGCGCACTGCTTCGAGAACGCCCACCGCGTGCGGGTGTCCGCCGACACCTGCAGTGTCTGTGAGGCCGCCTTGCTTGATGTGGACTTCAACAAGGCCAAGTCCCCACTCCCGGGCGATGAGACGCAGCACATGGGCTGCGTCTTTTGTGACCCCGTCTTCCAGGAGCTGGTGGAGCTGAAGCATGCGGCCTCCTGCCACCCCATGCACCGCGGTGGACCAGGGAGAAGGCAGGGTCGAGGGCGGGGCCGGGCCAGGAGGCCCCCTGGGAAGCCCAACCCCAGACGGCCCAAGGACAAGATGTCAGCCCTGGCCGCCTACTTTGTAAGCGGACCGACGCGTACGCGGCCGCTCGAGCAGAAACTCATCTCAGAAGAGGATCTGGCAGCAAATGATATCCTGgactacaaagaccatgacggtgattataaagatcatgacatcgattacaaggatgacgatgacaagGTTTAA

**TOP3B-3xFLAG R338W**

ATGAAGACTGTGCTCATGGTTGCTGAAAAGCCGTCCTTGGCACAGTCAATTGCCAAAATCCTCTCTAGAGGGAGCCTGTCCTCACACAAAGGGCTGAACGGGGCCTGCTCAGTCCACGAGTACACTGGGACCTTTGCTGGCCAGCCAGTGCGCTTCAAGATGACGTCTGTCTGTGGTCACGTGATGACCCTGGATTTCCTGGGAAAATACAACAAATGGGACAAAGTGGACCCCGCAGAACTGTTCAGCCAAGCTCCCACGGAGAAGAAAGAAGCTAACCCCAAGCTGAACATGGTGAAGTTCCTGCAGGTGGAGGGCAGAGGCTGCGACTACATCGTGCTGTGGCTGGACTGCGACAAGGAGGGGGAGAACATCTGCTTTGAGGTTCTTGATGCTGTTCTGCCCGTCATGAACAAGGCCCATGGTGGCGAGAAGACCGTGTTCCGGGCCAGGTTTAGCTCCATCACGGACACAGACATCTGTAATGCCATGGCCTGCCTAGGCGAGCCTGACCACAACGAGGCGCTCTCAGTGGATGCTCGCCAGGAGCTGGACCTGCGAATCGGCTGTGCATTCACCAGGTTTCAGACTAAATATTTCCAGGGGAAATACGGTGATTTAGACAGCTCTCTCATCTCCTTTGGGCCGTGTCAGACTCCAACCCTGGGATTCTGTGTGGAGAGACATGATAAAATCCAGTCCTTCAAACCAGAGACCTACTGGGTGCTGCAGGCCAAGGTTAACACTGACAAAGACAGATCTCTCCTTTTGGACTGGGACCGAGTAAGAGTGTTTGACCGGGAGATCGCACAGATGTTTTTAAACATGACAAAGCTGGAGAAGGAAGCCCAGGTGGAGGCCACAAGCAGGAAAGAAAAGGCCAAGCAGAGGCCCCTGGCCCTGAACACTGTGGAGATGCTGCGTGTGGCCAGCTCTTCTCTGGGCATGGGGCCGCAGCACGCCATGCAGACGGCTGAGCGGCTCTACACGCAAGGCTACATCAGCTACCCAtggACAGAGACCACCCACTACCCTGAGAACTTTGACCTGAAGGGCTCTCTGCGGCAGCAGGCCAACCACCCCTACTGGGCCGACACGGTGAAGCGGTTGTTAGCAGAAGGTATCAACCGCCCGCGGAAAGGCCATGACGCCGGCGACCATCCCCCCATCACCCCCATGAAGTCTGCCACAGAGGCCGAATTAGGGGGTGACGCGTGGCGGCTCTATGAGTACATCACCAGACACTTCATCGCCACGGTCAGCCATGACTGCAAGTACCTGCAGAGCACCATCTCCTTCAGAATTGGGCCCGAGCTCTTCACCTGCTCCGGGAAGACCGTCCTCTCACCAGGCTTCACGGAGGTCATGCCCTGGCAGAGCGTGCCCCTGGAGGAGAGCCTGCCCACTTGCCAGCGGGGTGATGCCTTCCCTGTGGGCGAGGTGAAGATGCTGGAGAAGCAGACGAACCCACCCGACTACCTGACGGAGGCCGAGCTCATCACGCTCATGGAGAAGCATGGCATCGGCACGGATGCCAGCATCCCTGTGCATATCAACAACATCTGCCAGCGCAACTATGTCACGGTGGAGAGCGGGCGCCGGCTCAAGCCCACCAACCTCGGCATCGTCCTGGTGCACGGCTACTATAAGATTGATGCAGAGCTGGTGCTCCCCACCATCCGCAGTGCAGTGGAGAAGCAGCTGAACCTGATCGCCCAGGGCAAGGCCGACTACCGCCAGGTCCTGGGCCACACCCTGGACGTGTTCAAGAGGAAGTTCCACTACTTTGTCGACTCCATTGCTGGCATGGATGAGTTGATGGAGGTGTCTTTCTCGCCCCTGGCGGCCACAGGCAAGCCCCTCTCACGCTGTGGGAAGTGCCACCGCTTCATGAAGTACATCCAGGCCAAGCCAAGCCGCCTGCACTGCTCCCACTGCGATGAGACCTACACGCTCCCCCAGAACGGCACCATCAAGCTCTACAAGGAGCTCCGCTGCCCTCTGGATGACTTCGAGCTGGTCCTGTGGTCATCAGGCTCTCGGGGCAAGAGCTACCCGCTGTGCCCCTACTGCTACAACCACCCACCCTTCCGAGACATGAAGAAAGGCATGGGCTGCAACGAGTGTACGCACCCCTCCTGCCAGCACTCGCTGAGCATGCTGGGCATCGGCCAGTGCGTGGAATGTGAGAGCGGGGTGCTGGTGCTGGACCCCACCTCGGGCCCCAAGTGGAAGGTGGCCTGCAACAAGTGCAACGTGGTAGCGCACTGCTTCGAGAACGCCCACCGCGTGCGGGTGTCCGCCGACACCTGCAGTGTCTGTGAGGCCGCCTTGCTTGATGTGGACTTCAACAAGGCCAAGTCCCCACTCCCGGGCGATGAGACGCAGCACATGGGCTGCGTCTTTTGTGACCCCGTCTTCCAGGAGCTGGTGGAGCTGAAGCATGCGGCCTCCTGCCACCCCATGCACCGCGGTGGACCAGGGAGAAGGCAGGGTCGAGGGCGGGGCCGGGCCAGGAGGCCCCCTGGGAAGCCCAACCCCAGACGGCCCAAGGACAAGATGTCAGCCCTGGCCGCCTACTTTGTAAGCGGACCGACGCGTACGCGGCCGCTCGAGCAGAAACTCATCTCAGAAGAGGATCTGGCAGCAAATGATATCCTGgactacaaagaccatgacggtgattataaagatcatgacatcgattacaaggatgacgatgacaagGTTTAA

**TOP3B-3xFLAG P378Q**

ATGAAGACTGTGCTCATGGTTGCTGAAAAGCCGTCCTTGGCACAGTCAATTGCCAAAATCCTCTCTAGAGGGAGCCTGTCCTCACACAAAGGGCTGAACGGGGCCTGCTCAGTCCACGAGTACACTGGGACCTTTGCTGGCCAGCCAGTGCGCTTCAAGATGACGTCTGTCTGTGGTCACGTGATGACCCTGGATTTCCTGGGAAAATACAACAAATGGGACAAAGTGGACCCCGCAGAACTGTTCAGCCAAGCTCCCACGGAGAAGAAAGAAGCTAACCCCAAGCTGAACATGGTGAAGTTCCTGCAGGTGGAGGGCAGAGGCTGCGACTACATCGTGCTGTGGCTGGACTGCGACAAGGAGGGGGAGAACATCTGCTTTGAGGTTCTTGATGCTGTTCTGCCCGTCATGAACAAGGCCCATGGTGGCGAGAAGACCGTGTTCCGGGCCAGGTTTAGCTCCATCACGGACACAGACATCTGTAATGCCATGGCCTGCCTAGGCGAGCCTGACCACAACGAGGCGCTCTCAGTGGATGCTCGCCAGGAGCTGGACCTGCGAATCGGCTGTGCATTCACCAGGTTTCAGACTAAATATTTCCAGGGGAAATACGGTGATTTAGACAGCTCTCTCATCTCCTTTGGGCCGTGTCAGACTCCAACCCTGGGATTCTGTGTGGAGAGACATGATAAAATCCAGTCCTTCAAACCAGAGACCTACTGGGTGCTGCAGGCCAAGGTTAACACTGACAAAGACAGATCTCTCCTTTTGGACTGGGACCGAGTAAGAGTGTTTGACCGGGAGATCGCACAGATGTTTTTAAACATGACAAAGCTGGAGAAGGAAGCCCAGGTGGAGGCCACAAGCAGGAAAGAAAAGGCCAAGCAGAGGCCCCTGGCCCTGAACACTGTGGAGATGCTGCGTGTGGCCAGCTCTTCTCTGGGCATGGGGCCGCAGCACGCCATGCAGACGGCTGAGCGGCTCTACACGCAAGGCTACATCAGCTACCCACGGACAGAGACCACCCACTACCCTGAGAACTTTGACCTGAAGGGCTCTCTGCGGCAGCAGGCCAACCACCCCTACTGGGCCGACACGGTGAAGCGGTTGTTAGCAGAAGGTATCAACCGCcagCGGAAAGGCCATGACGCCGGCGACCATCCCCCCATCACCCCCATGAAGTCTGCCACAGAGGCCGAATTAGGGGGTGACGCGTGGCGGCTCTATGAGTACATCACCAGACACTTCATCGCCACGGTCAGCCATGACTGCAAGTACCTGCAGAGCACCATCTCCTTCAGAATTGGGCCCGAGCTCTTCACCTGCTCCGGGAAGACCGTCCTCTCACCAGGCTTCACGGAGGTCATGCCCTGGCAGAGCGTGCCCCTGGAGGAGAGCCTGCCCACTTGCCAGCGGGGTGATGCCTTCCCTGTGGGCGAGGTGAAGATGCTGGAGAAGCAGACGAACCCACCCGACTACCTGACGGAGGCCGAGCTCATCACGCTCATGGAGAAGCATGGCATCGGCACGGATGCCAGCATCCCTGTGCATATCAACAACATCTGCCAGCGCAACTATGTCACGGTGGAGAGCGGGCGCCGGCTCAAGCCCACCAACCTCGGCATCGTCCTGGTGCACGGCTACTATAAGATTGATGCAGAGCTGGTGCTCCCCACCATCCGCAGTGCAGTGGAGAAGCAGCTGAACCTGATCGCCCAGGGCAAGGCCGACTACCGCCAGGTCCTGGGCCACACCCTGGACGTGTTCAAGAGGAAGTTCCACTACTTTGTCGACTCCATTGCTGGCATGGATGAGTTGATGGAGGTGTCTTTCTCGCCCCTGGCGGCCACAGGCAAGCCCCTCTCACGCTGTGGGAAGTGCCACCGCTTCATGAAGTACATCCAGGCCAAGCCAAGCCGCCTGCACTGCTCCCACTGCGATGAGACCTACACGCTCCCCCAGAACGGCACCATCAAGCTCTACAAGGAGCTCCGCTGCCCTCTGGATGACTTCGAGCTGGTCCTGTGGTCATCAGGCTCTCGGGGCAAGAGCTACCCGCTGTGCCCCTACTGCTACAACCACCCACCCTTCCGAGACATGAAGAAAGGCATGGGCTGCAACGAGTGTACGCACCCCTCCTGCCAGCACTCGCTGAGCATGCTGGGCATCGGCCAGTGCGTGGAATGTGAGAGCGGGGTGCTGGTGCTGGACCCCACCTCGGGCCCCAAGTGGAAGGTGGCCTGCAACAAGTGCAACGTGGTAGCGCACTGCTTCGAGAACGCCCACCGCGTGCGGGTGTCCGCCGACACCTGCAGTGTCTGTGAGGCCGCCTTGCTTGATGTGGACTTCAACAAGGCCAAGTCCCCACTCCCGGGCGATGAGACGCAGCACATGGGCTGCGTCTTTTGTGACCCCGTCTTCCAGGAGCTGGTGGAGCTGAAGCATGCGGCCTCCTGCCACCCCATGCACCGCGGTGGACCAGGGAGAAGGCAGGGTCGAGGGCGGGGCCGGGCCAGGAGGCCCCCTGGGAAGCCCAACCCCAGACGGCCCAAGGACAAGATGTCAGCCCTGGCCGCCTACTTTGTAAGCGGACCGACGCGTACGCGGCCGCTCGAGCAGAAACTCATCTCAGAAGAGGATCTGGCAGCAAATGATATCCTGgactacaaagaccatgacggtgattataaagatcatgacatcgattacaaggatgacgatgacaagGTTTAA

**TOP3B-3xFLAG R472Q**

ATGAAGACTGTGCTCATGGTTGCTGAAAAGCCGTCCTTGGCACAGTCAATTGCCAAAATCCTCTCTAGAGGGAGCCTGTCCTCACACAAAGGGCTGAACGGGGCCTGCTCAGTCCACGAGTACACTGGGACCTTTGCTGGCCAGCCAGTGCGCTTCAAGATGACGTCTGTCTGTGGTCACGTGATGACCCTGGATTTCCTGGGAAAATACAACAAATGGGACAAAGTGGACCCCGCAGAACTGTTCAGCCAAGCTCCCACGGAGAAGAAAGAAGCTAACCCCAAGCTGAACATGGTGAAGTTCCTGCAGGTGGAGGGCAGAGGCTGCGACTACATCGTGCTGTGGCTGGACTGCGACAAGGAGGGGGAGAACATCTGCTTTGAGGTTCTTGATGCTGTTCTGCCCGTCATGAACAAGGCCCATGGTGGCGAGAAGACCGTGTTCCGGGCCAGGTTTAGCTCCATCACGGACACAGACATCTGTAATGCCATGGCCTGCCTAGGCGAGCCTGACCACAACGAGGCGCTCTCAGTGGATGCTCGCCAGGAGCTGGACCTGCGAATCGGCTGTGCATTCACCAGGTTTCAGACTAAATATTTCCAGGGGAAATACGGTGATTTAGACAGCTCTCTCATCTCCTTTGGGCCGTGTCAGACTCCAACCCTGGGATTCTGTGTGGAGAGACATGATAAAATCCAGTCCTTCAAACCAGAGACCTACTGGGTGCTGCAGGCCAAGGTTAACACTGACAAAGACAGATCTCTCCTTTTGGACTGGGACCGAGTAAGAGTGTTTGACCGGGAGATCGCACAGATGTTTTTAAACATGACAAAGCTGGAGAAGGAAGCCCAGGTGGAGGCCACAAGCAGGAAAGAAAAGGCCAAGCAGAGGCCCCTGGCCCTGAACACTGTGGAGATGCTGCGTGTGGCCAGCTCTTCTCTGGGCATGGGGCCGCAGCACGCCATGCAGACGGCTGAGCGGCTCTACACGCAAGGCTACATCAGCTACCCACGGACAGAGACCACCCACTACCCTGAGAACTTTGACCTGAAGGGCTCTCTGCGGCAGCAGGCCAACCACCCCTACTGGGCCGACACGGTGAAGCGGTTGTTAGCAGAAGGTATCAACCGCCCGCGGAAAGGCCATGACGCCGGCGACCATCCCCCCATCACCCCCATGAAGTCTGCCACAGAGGCCGAATTAGGGGGTGACGCGTGGCGGCTCTATGAGTACATCACCAGACACTTCATCGCCACGGTCAGCCATGACTGCAAGTACCTGCAGAGCACCATCTCCTTCAGAATTGGGCCCGAGCTCTTCACCTGCTCCGGGAAGACCGTCCTCTCACCAGGCTTCACGGAGGTCATGCCCTGGCAGAGCGTGCCCCTGGAGGAGAGCCTGCCCACTTGCCAGcagGGTGATGCCTTCCCTGTGGGCGAGGTGAAGATGCTGGAGAAGCAGACGAACCCACCCGACTACCTGACGGAGGCCGAGCTCATCACGCTCATGGAGAAGCATGGCATCGGCACGGATGCCAGCATCCCTGTGCATATCAACAACATCTGCCAGCGCAACTATGTCACGGTGGAGAGCGGGCGCCGGCTCAAGCCCACCAACCTCGGCATCGTCCTGGTGCACGGCTACTATAAGATTGATGCAGAGCTGGTGCTCCCCACCATCCGCAGTGCAGTGGAGAAGCAGCTGAACCTGATCGCCCAGGGCAAGGCCGACTACCGCCAGGTCCTGGGCCACACCCTGGACGTGTTCAAGAGGAAGTTCCACTACTTTGTCGACTCCATTGCTGGCATGGATGAGTTGATGGAGGTGTCTTTCTCGCCCCTGGCGGCCACAGGCAAGCCCCTCTCACGCTGTGGGAAGTGCCACCGCTTCATGAAGTACATCCAGGCCAAGCCAAGCCGCCTGCACTGCTCCCACTGCGATGAGACCTACACGCTCCCCCAGAACGGCACCATCAAGCTCTACAAGGAGCTCCGCTGCCCTCTGGATGACTTCGAGCTGGTCCTGTGGTCATCAGGCTCTCGGGGCAAGAGCTACCCGCTGTGCCCCTACTGCTACAACCACCCACCCTTCCGAGACATGAAGAAAGGCATGGGCTGCAACGAGTGTACGCACCCCTCCTGCCAGCACTCGCTGAGCATGCTGGGCATCGGCCAGTGCGTGGAATGTGAGAGCGGGGTGCTGGTGCTGGACCCCACCTCGGGCCCCAAGTGGAAGGTGGCCTGCAACAAGTGCAACGTGGTAGCGCACTGCTTCGAGAACGCCCACCGCGTGCGGGTGTCCGCCGACACCTGCAGTGTCTGTGAGGCCGCCTTGCTTGATGTGGACTTCAACAAGGCCAAGTCCCCACTCCCGGGCGATGAGACGCAGCACATGGGCTGCGTCTTTTGTGACCCCGTCTTCCAGGAGCTGGTGGAGCTGAAGCATGCGGCCTCCTGCCACCCCATGCACCGCGGTGGACCAGGGAGAAGGCAGGGTCGAGGGCGGGGCCGGGCCAGGAGGCCCCCTGGGAAGCCCAACCCCAGACGGCCCAAGGACAAGATGTCAGCCCTGGCCGCCTACTTTGTAAGCGGACCGACGCGTACGCGGCCGCTCGAGCAGAAACTCATCTCAGAAGAGGATCTGGCAGCAAATGATATCCTGgactacaaagaccatgacggtgattataaagatcatgacatcgattacaaggatgacgatgacaagGTTTAA

**TOP3B-3xFLAG C666R**

ATGAAGACTGTGCTCATGGTTGCTGAAAAGCCGTCCTTGGCACAGTCAATTGCCAAAATCCTCTCTAGAGGGAGCCTGTCCTCACACAAAGGGCTGAACGGGGCCTGCTCAGTCCACGAGTACACTGGGACCTTTGCTGGCCAGCCAGTGCGCTTCAAGATGACGTCTGTCTGTGGTCACGTGATGACCCTGGATTTCCTGGGAAAATACAACAAATGGGACAAAGTGGACCCCGCAGAACTGTTCAGCCAAGCTCCCACGGAGAAGAAAGAAGCTAACCCCAAGCTGAACATGGTGAAGTTCCTGCAGGTGGAGGGCAGAGGCTGCGACTACATCGTGCTGTGGCTGGACTGCGACAAGGAGGGGGAGAACATCTGCTTTGAGGTTCTTGATGCTGTTCTGCCCGTCATGAACAAGGCCCATGGTGGCGAGAAGACCGTGTTCCGGGCCAGGTTTAGCTCCATCACGGACACAGACATCTGTAATGCCATGGCCTGCCTAGGCGAGCCTGACCACAACGAGGCGCTCTCAGTGGATGCTCGCCAGGAGCTGGACCTGCGAATCGGCTGTGCATTCACCAGGTTTCAGACTAAATATTTCCAGGGGAAATACGGTGATTTAGACAGCTCTCTCATCTCCTTTGGGCCGTGTCAGACTCCAACCCTGGGATTCTGTGTGGAGAGACATGATAAAATCCAGTCCTTCAAACCAGAGACCTACTGGGTGCTGCAGGCCAAGGTTAACACTGACAAAGACAGATCTCTCCTTTTGGACTGGGACCGAGTAAGAGTGTTTGACCGGGAGATCGCACAGATGTTTTTAAACATGACAAAGCTGGAGAAGGAAGCCCAGGTGGAGGCCACAAGCAGGAAAGAAAAGGCCAAGCAGAGGCCCCTGGCCCTGAACACTGTGGAGATGCTGCGTGTGGCCAGCTCTTCTCTGGGCATGGGGCCGCAGCACGCCATGCAGACGGCTGAGCGGCTCTACACGCAAGGCTACATCAGCTACCCACGGACAGAGACCACCCACTACCCTGAGAACTTTGACCTGAAGGGCTCTCTGCGGCAGCAGGCCAACCACCCCTACTGGGCCGACACGGTGAAGCGGTTGTTAGCAGAAGGTATCAACCGCCCGCGGAAAGGCCATGACGCCGGCGACCATCCCCCCATCACCCCCATGAAGTCTGCCACAGAGGCCGAATTAGGGGGTGACGCGTGGCGGCTCTATGAGTACATCACCAGACACTTCATCGCCACGGTCAGCCATGACTGCAAGTACCTGCAGAGCACCATCTCCTTCAGAATTGGGCCCGAGCTCTTCACCTGCTCCGGGAAGACCGTCCTCTCACCAGGCTTCACGGAGGTCATGCCCTGGCAGAGCGTGCCCCTGGAGGAGAGCCTGCCCACTTGCCAGCGGGGTGATGCCTTCCCTGTGGGCGAGGTGAAGATGCTGGAGAAGCAGACGAACCCACCCGACTACCTGACGGAGGCCGAGCTCATCACGCTCATGGAGAAGCATGGCATCGGCACGGATGCCAGCATCCCTGTGCATATCAACAACATCTGCCAGCGCAACTATGTCACGGTGGAGAGCGGGCGCCGGCTCAAGCCCACCAACCTCGGCATCGTCCTGGTGCACGGCTACTATAAGATTGATGCAGAGCTGGTGCTCCCCACCATCCGCAGTGCAGTGGAGAAGCAGCTGAACCTGATCGCCCAGGGCAAGGCCGACTACCGCCAGGTCCTGGGCCACACCCTGGACGTGTTCAAGAGGAAGTTCCACTACTTTGTCGACTCCATTGCTGGCATGGATGAGTTGATGGAGGTGTCTTTCTCGCCCCTGGCGGCCACAGGCAAGCCCCTCTCACGCTGTGGGAAGTGCCACCGCTTCATGAAGTACATCCAGGCCAAGCCAAGCCGCCTGCACTGCTCCCACTGCGATGAGACCTACACGCTCCCCCAGAACGGCACCATCAAGCTCTACAAGGAGCTCCGCcgcCCTCTGGATGACTTCGAGCTGGTCCTGTGGTCATCAGGCTCTCGGGGCAAGAGCTACCCGCTGTGCCCCTACTGCTACAACCACCCACCCTTCCGAGACATGAAGAAAGGCATGGGCTGCAACGAGTGTACGCACCCCTCCTGCCAGCACTCGCTGAGCATGCTGGGCATCGGCCAGTGCGTGGAATGTGAGAGCGGGGTGCTGGTGCTGGACCCCACCTCGGGCCCCAAGTGGAAGGTGGCCTGCAACAAGTGCAACGTGGTAGCGCACTGCTTCGAGAACGCCCACCGCGTGCGGGTGTCCGCCGACACCTGCAGTGTCTGTGAGGCCGCCTTGCTTGATGTGGACTTCAACAAGGCCAAGTCCCCACTCCCGGGCGATGAGACGCAGCACATGGGCTGCGTCTTTTGTGACCCCGTCTTCCAGGAGCTGGTGGAGCTGAAGCATGCGGCCTCCTGCCACCCCATGCACCGCGGTGGACCAGGGAGAAGGCAGGGTCGAGGGCGGGGCCGGGCCAGGAGGCCCCCTGGGAAGCCCAACCCCAGACGGCCCAAGGACAAGATGTCAGCCCTGGCCGCCTACTTTGTAAGCGGACCGACGCGTACGCGGCCGCTCGAGCAGAAACTCATCTCAGAAGAGGATCTGGCAGCAAATGATATCCTGgactacaaagaccatgacggtgattataaagatcatgacatcgattacaaggatgacgatgacaagGTTTAA

**3xFLAG-TOP3B WT**

ATGggttcttctgactacaaagaccatgacggtgattataaagatcatgacatcgattacaaggatgacgatgacaagggttcttctAAGACTGTGCTCATGGTTGCTGAAAAGCCGTCCTTGGCACAGTCAATTGCCAAAATCCTCTCTAGAGGGAGCCTGTCCTCACACAAAGGGCTGAACGGGGCCTGCTCAGTCCACGAGTACACTGGGACCTTTGCTGGCCAGCCAGTGCGCTTCAAGATGACGTCTGTCTGTGGTCACGTGATGACCCTGGATTTCCTGGGAAAATACAACAAATGGGACAAAGTGGACCCCGCAGAACTGTTCAGCCAAGCTCCCACGGAGAAGAAAGAAGCTAACCCCAAGCTGAACATGGTGAAGTTCCTGCAGGTGGAGGGCAGAGGCTGCGACTACATCGTGCTGTGGCTGGACTGCGACAAGGAGGGGGAGAACATCTGCTTTGAGGTTCTTGATGCTGTTCTGCCCGTCATGAACAAGGCCCATGGTGGCGAGAAGACCGTGTTCCGGGCCAGGTTTAGCTCCATCACGGACACAGACATCTGTAATGCCATGGCCTGCCTAGGCGAGCCTGACCACAACGAGGCGCTCTCAGTGGATGCTCGCCAGGAGCTGGACCTGCGAATCGGCTGTGCATTCACCAGGTTTCAGACTAAATATTTCCAGGGGAAATACGGTGATTTAGACAGCTCTCTCATCTCCTTTGGGCCGTGTCAGACTCCAACCCTGGGATTCTGTGTGGAGAGACATGATAAAATCCAGTCCTTCAAACCAGAGACCTACTGGGTGCTGCAGGCCAAGGTTAACACTGACAAAGACAGATCTCTCCTTTTGGACTGGGACCGAGTAAGAGTGTTTGACCGGGAGATCGCACAGATGTTTTTAAACATGACAAAGCTGGAGAAGGAAGCCCAGGTGGAGGCCACAAGCAGGAAAGAAAAGGCCAAGCAGAGGCCCCTGGCCCTGAACACTGTGGAGATGCTGCGTGTGGCCAGCTCTTCTCTGGGCATGGGGCCGCAGCACGCCATGCAGACGGCTGAGCGGCTCTACACGCAAGGCTACATCAGCTACCCACGGACAGAGACCACCCACTACCCTGAGAACTTTGACCTGAAGGGCTCTCTGCGGCAGCAGGCCAACCACCCCTACTGGGCCGACACGGTGAAGCGGTTGTTAGCAGAAGGTATCAACCGCCCGCGGAAAGGCCATGACGCCGGCGACCATCCCCCCATCACCCCCATGAAGTCTGCCACAGAGGCCGAATTAGGGGGTGACGCGTGGCGGCTCTATGAGTACATCACCAGACACTTCATCGCCACGGTCAGCCATGACTGCAAGTACCTGCAGAGCACCATCTCCTTCAGAATTGGGCCCGAGCTCTTCACCTGCTCCGGGAAGACCGTCCTCTCACCAGGCTTCACGGAGGTCATGCCCTGGCAGAGCGTGCCCCTGGAGGAGAGCCTGCCCACTTGCCAGCGGGGTGATGCCTTCCCTGTGGGCGAGGTGAAGATGCTGGAGAAGCAGACGAACCCACCCGACTACCTGACGGAGGCCGAGCTCATCACGCTCATGGAGAAGCATGGCATCGGCACGGATGCCAGCATCCCTGTGCATATCAACAACATCTGCCAGCGCAACTATGTCACGGTGGAGAGCGGGCGCCGGCTCAAGCCCACCAACCTCGGCATCGTCCTGGTGCACGGCTACTATAAGATTGATGCAGAGCTGGTGCTCCCCACCATCCGCAGTGCAGTGGAGAAGCAGCTGAACCTGATCGCCCAGGGCAAGGCCGACTACCGCCAGGTCCTGGGCCACACCCTGGACGTGTTCAAGAGGAAGTTCCACTACTTTGTCGACTCCATTGCTGGCATGGATGAGTTGATGGAGGTGTCTTTCTCGCCCCTGGCGGCCACAGGCAAGCCCCTCTCACGCTGTGGGAAGTGCCACCGCTTCATGAAGTACATCCAGGCCAAGCCAAGCCGCCTGCACTGCTCCCACTGCGATGAGACCTACACGCTCCCCCAGAACGGCACCATCAAGCTCTACAAGGAGCTCCGCTGCCCTCTGGATGACTTCGAGCTGGTCCTGTGGTCATCAGGCTCTCGGGGCAAGAGCTACCCGCTGTGCCCCTACTGCTACAACCACCCACCCTTCCGAGACATGAAGAAAGGCATGGGCTGCAACGAGTGTACGCACCCCTCCTGCCAGCACTCGCTGAGCATGCTGGGCATCGGCCAGTGCGTGGAATGTGAGAGCGGGGTGCTGGTGCTGGACCCCACCTCGGGCCCCAAGTGGAAGGTGGCCTGCAACAAGTGCAACGTGGTAGCGCACTGCTTCGAGAACGCCCACCGCGTGCGGGTGTCCGCCGACACCTGCAGTGTCTGTGAGGCCGCCTTGCTTGATGTGGACTTCAACAAGGCCAAGTCCCCACTCCCGGGCGATGAGACGCAGCACATGGGCTGCGTCTTTTGTGACCCCGTCTTCCAGGAGCTGGTGGAGCTGAAGCATGCGGCCTCCTGCCACCCCATGCACCGCGGTGGACCAGGGAGAAGGCAGGGTCGAGGGCGGGGCCGGGCCAGGAGGCCCCCTGGGAAGCCCAACCCCAGACGGCCCAAGGACAAGATGTCAGCCCTGGCCGCCTACTTTGTATAA

**3xFLAG-TOP3B Y336F**

ATGggttcttctgactacaaagaccatgacggtgattataaagatcatgacatcgattacaaggatgacgatgacaagggttcttctAAGACTGTGCTCATGGTTGCTGAAAAGCCGTCCTTGGCACAGTCAATTGCCAAAATCCTCTCTAGAGGGAGCCTGTCCTCACACAAAGGGCTGAACGGGGCCTGCTCAGTCCACGAGTACACTGGGACCTTTGCTGGCCAGCCAGTGCGCTTCAAGATGACGTCTGTCTGTGGTCACGTGATGACCCTGGATTTCCTGGGAAAATACAACAAATGGGACAAAGTGGACCCCGCAGAACTGTTCAGCCAAGCTCCCACGGAGAAGAAAGAAGCTAACCCCAAGCTGAACATGGTGAAGTTCCTGCAGGTGGAGGGCAGAGGCTGCGACTACATCGTGCTGTGGCTGGACTGCGACAAGGAGGGGGAGAACATCTGCTTTGAGGTTCTTGATGCTGTTCTGCCCGTCATGAACAAGGCCCATGGTGGCGAGAAGACCGTGTTCCGGGCCAGGTTTAGCTCCATCACGGACACAGACATCTGTAATGCCATGGCCTGCCTAGGCGAGCCTGACCACAACGAGGCGCTCTCAGTGGATGCTCGCCAGGAGCTGGACCTGCGAATCGGCTGTGCATTCACCAGGTTTCAGACTAAATATTTCCAGGGGAAATACGGTGATTTAGACAGCTCTCTCATCTCCTTTGGGCCGTGTCAGACTCCAACCCTGGGATTCTGTGTGGAGAGACATGATAAAATCCAGTCCTTCAAACCAGAGACCTACTGGGTGCTGCAGGCCAAGGTTAACACTGACAAAGACAGATCTCTCCTTTTGGACTGGGACCGAGTAAGAGTGTTTGACCGGGAGATCGCACAGATGTTTTTAAACATGACAAAGCTGGAGAAGGAAGCCCAGGTGGAGGCCACAAGCAGGAAAGAAAAGGCCAAGCAGAGGCCCCTGGCCCTGAACACTGTGGAGATGCTGCGTGTGGCCAGCTCTTCTCTGGGCATGGGGCCGCAGCACGCCATGCAGACGGCTGAGCGGCTCTACACGCAAGGCTACATCAGCtttCCACGGACAGAGACCACCCACTACCCTGAGAACTTTGACCTGAAGGGCTCTCTGCGGCAGCAGGCCAACCACCCCTACTGGGCCGACACGGTGAAGCGGTTGTTAGCAGAAGGTATCAACCGCCCGCGGAAAGGCCATGACGCCGGCGACCATCCCCCCATCACCCCCATGAAGTCTGCCACAGAGGCCGAATTAGGGGGTGACGCGTGGCGGCTCTATGAGTACATCACCAGACACTTCATCGCCACGGTCAGCCATGACTGCAAGTACCTGCAGAGCACCATCTCCTTCAGAATTGGGCCCGAGCTCTTCACCTGCTCCGGGAAGACCGTCCTCTCACCAGGCTTCACGGAGGTCATGCCCTGGCAGAGCGTGCCCCTGGAGGAGAGCCTGCCCACTTGCCAGCGGGGTGATGCCTTCCCTGTGGGCGAGGTGAAGATGCTGGAGAAGCAGACGAACCCACCCGACTACCTGACGGAGGCCGAGCTCATCACGCTCATGGAGAAGCATGGCATCGGCACGGATGCCAGCATCCCTGTGCATATCAACAACATCTGCCAGCGCAACTATGTCACGGTGGAGAGCGGGCGCCGGCTCAAGCCCACCAACCTCGGCATCGTCCTGGTGCACGGCTACTATAAGATTGATGCAGAGCTGGTGCTCCCCACCATCCGCAGTGCAGTGGAGAAGCAGCTGAACCTGATCGCCCAGGGCAAGGCCGACTACCGCCAGGTCCTGGGCCACACCCTGGACGTGTTCAAGAGGAAGTTCCACTACTTTGTCGACTCCATTGCTGGCATGGATGAGTTGATGGAGGTGTCTTTCTCGCCCCTGGCGGCCACAGGCAAGCCCCTCTCACGCTGTGGGAAGTGCCACCGCTTCATGAAGTACATCCAGGCCAAGCCAAGCCGCCTGCACTGCTCCCACTGCGATGAGACCTACACGCTCCCCCAGAACGGCACCATCAAGCTCTACAAGGAGCTCCGCTGCCCTCTGGATGACTTCGAGCTGGTCCTGTGGTCATCAGGCTCTCGGGGCAAGAGCTACCCGCTGTGCCCCTACTGCTACAACCACCCACCCTTCCGAGACATGAAGAAAGGCATGGGCTGCAACGAGTGTACGCACCCCTCCTGCCAGCACTCGCTGAGCATGCTGGGCATCGGCCAGTGCGTGGAATGTGAGAGCGGGGTGCTGGTGCTGGACCCCACCTCGGGCCCCAAGTGGAAGGTGGCCTGCAACAAGTGCAACGTGGTAGCGCACTGCTTCGAGAACGCCCACCGCGTGCGGGTGTCCGCCGACACCTGCAGTGTCTGTGAGGCCGCCTTGCTTGATGTGGACTTCAACAAGGCCAAGTCCCCACTCCCGGGCGATGAGACGCAGCACATGGGCTGCGTCTTTTGTGACCCCGTCTTCCAGGAGCTGGTGGAGCTGAAGCATGCGGCCTCCTGCCACCCCATGCACCGCGGTGGACCAGGGAGAAGGCAGGGTCGAGGGCGGGGCCGGGCCAGGAGGCCCCCTGGGAAGCCCAACCCCAGACGGCCCAAGGACAAGATGTCAGCCCTGGCCGCCTACTTTGTATAA

**3xFLAG-TOP3B R338W**

ATGggttcttctgactacaaagaccatgacggtgattataaagatcatgacatcgattacaaggatgacgatgacaagggttcttctAAGACTGTGCTCATGGTTGCTGAAAAGCCGTCCTTGGCACAGTCAATTGCCAAAATCCTCTCTAGAGGGAGCCTGTCCTCACACAAAGGGCTGAACGGGGCCTGCTCAGTCCACGAGTACACTGGGACCTTTGCTGGCCAGCCAGTGCGCTTCAAGATGACGTCTGTCTGTGGTCACGTGATGACCCTGGATTTCCTGGGAAAATACAACAAATGGGACAAAGTGGACCCCGCAGAACTGTTCAGCCAAGCTCCCACGGAGAAGAAAGAAGCTAACCCCAAGCTGAACATGGTGAAGTTCCTGCAGGTGGAGGGCAGAGGCTGCGACTACATCGTGCTGTGGCTGGACTGCGACAAGGAGGGGGAGAACATCTGCTTTGAGGTTCTTGATGCTGTTCTGCCCGTCATGAACAAGGCCCATGGTGGCGAGAAGACCGTGTTCCGGGCCAGGTTTAGCTCCATCACGGACACAGACATCTGTAATGCCATGGCCTGCCTAGGCGAGCCTGACCACAACGAGGCGCTCTCAGTGGATGCTCGCCAGGAGCTGGACCTGCGAATCGGCTGTGCATTCACCAGGTTTCAGACTAAATATTTCCAGGGGAAATACGGTGATTTAGACAGCTCTCTCATCTCCTTTGGGCCGTGTCAGACTCCAACCCTGGGATTCTGTGTGGAGAGACATGATAAAATCCAGTCCTTCAAACCAGAGACCTACTGGGTGCTGCAGGCCAAGGTTAACACTGACAAAGACAGATCTCTCCTTTTGGACTGGGACCGAGTAAGAGTGTTTGACCGGGAGATCGCACAGATGTTTTTAAACATGACAAAGCTGGAGAAGGAAGCCCAGGTGGAGGCCACAAGCAGGAAAGAAAAGGCCAAGCAGAGGCCCCTGGCCCTGAACACTGTGGAGATGCTGCGTGTGGCCAGCTCTTCTCTGGGCATGGGGCCGCAGCACGCCATGCAGACGGCTGAGCGGCTCTACACGCAAGGCTACATCAGCTACCCAtggACAGAGACCACCCACTACCCTGAGAACTTTGACCTGAAGGGCTCTCTGCGGCAGCAGGCCAACCACCCCTACTGGGCCGACACGGTGAAGCGGTTGTTAGCAGAAGGTATCAACCGCCCGCGGAAAGGCCATGACGCCGGCGACCATCCCCCCATCACCCCCATGAAGTCTGCCACAGAGGCCGAATTAGGGGGTGACGCGTGGCGGCTCTATGAGTACATCACCAGACACTTCATCGCCACGGTCAGCCATGACTGCAAGTACCTGCAGAGCACCATCTCCTTCAGAATTGGGCCCGAGCTCTTCACCTGCTCCGGGAAGACCGTCCTCTCACCAGGCTTCACGGAGGTCATGCCCTGGCAGAGCGTGCCCCTGGAGGAGAGCCTGCCCACTTGCCAGCGGGGTGATGCCTTCCCTGTGGGCGAGGTGAAGATGCTGGAGAAGCAGACGAACCCACCCGACTACCTGACGGAGGCCGAGCTCATCACGCTCATGGAGAAGCATGGCATCGGCACGGATGCCAGCATCCCTGTGCATATCAACAACATCTGCCAGCGCAACTATGTCACGGTGGAGAGCGGGCGCCGGCTCAAGCCCACCAACCTCGGCATCGTCCTGGTGCACGGCTACTATAAGATTGATGCAGAGCTGGTGCTCCCCACCATCCGCAGTGCAGTGGAGAAGCAGCTGAACCTGATCGCCCAGGGCAAGGCCGACTACCGCCAGGTCCTGGGCCACACCCTGGACGTGTTCAAGAGGAAGTTCCACTACTTTGTCGACTCCATTGCTGGCATGGATGAGTTGATGGAGGTGTCTTTCTCGCCCCTGGCGGCCACAGGCAAGCCCCTCTCACGCTGTGGGAAGTGCCACCGCTTCATGAAGTACATCCAGGCCAAGCCAAGCCGCCTGCACTGCTCCCACTGCGATGAGACCTACACGCTCCCCCAGAACGGCACCATCAAGCTCTACAAGGAGCTCCGCTGCCCTCTGGATGACTTCGAGCTGGTCCTGTGGTCATCAGGCTCTCGGGGCAAGAGCTACCCGCTGTGCCCCTACTGCTACAACCACCCACCCTTCCGAGACATGAAGAAAGGCATGGGCTGCAACGAGTGTACGCACCCCTCCTGCCAGCACTCGCTGAGCATGCTGGGCATCGGCCAGTGCGTGGAATGTGAGAGCGGGGTGCTGGTGCTGGACCCCACCTCGGGCCCCAAGTGGAAGGTGGCCTGCAACAAGTGCAACGTGGTAGCGCACTGCTTCGAGAACGCCCACCGCGTGCGGGTGTCCGCCGACACCTGCAGTGTCTGTGAGGCCGCCTTGCTTGATGTGGACTTCAACAAGGCCAAGTCCCCACTCCCGGGCGATGAGACGCAGCACATGGGCTGCGTCTTTTGTGACCCCGTCTTCCAGGAGCTGGTGGAGCTGAAGCATGCGGCCTCCTGCCACCCCATGCACCGCGGTGGACCAGGGAGAAGGCAGGGTCGAGGGCGGGGCCGGGCCAGGAGGCCCCCTGGGAAGCCCAACCCCAGACGGCCCAAGGACAAGATGTCAGCCCTGGCCGCCTACTTTGTATAA

**3xFLAG-TOP3B R338W 1-823**

ATGggttcttctgactacaaagaccatgacggtgattataaagatcatgacatcgattacaaggatgacgatgacaagggttcttctAAGACTGTGCTCATGGTTGCTGAAAAGCCGTCCTTGGCACAGTCAATTGCCAAAATCCTCTCTAGAGGGAGCCTGTCCTCACACAAAGGGCTGAACGGGGCCTGCTCAGTCCACGAGTACACTGGGACCTTTGCTGGCCAGCCAGTGCGCTTCAAGATGACGTCTGTCTGTGGTCACGTGATGACCCTGGATTTCCTGGGAAAATACAACAAATGGGACAAAGTGGACCCCGCAGAACTGTTCAGCCAAGCTCCCACGGAGAAGAAAGAAGCTAACCCCAAGCTGAACATGGTGAAGTTCCTGCAGGTGGAGGGCAGAGGCTGCGACTACATCGTGCTGTGGCTGGACTGCGACAAGGAGGGGGAGAACATCTGCTTTGAGGTTCTTGATGCTGTTCTGCCCGTCATGAACAAGGCCCATGGTGGCGAGAAGACCGTGTTCCGGGCCAGGTTTAGCTCCATCACGGACACAGACATCTGTAATGCCATGGCCTGCCTAGGCGAGCCTGACCACAACGAGGCGCTCTCAGTGGATGCTCGCCAGGAGCTGGACCTGCGAATCGGCTGTGCATTCACCAGGTTTCAGACTAAATATTTCCAGGGGAAATACGGTGATTTAGACAGCTCTCTCATCTCCTTTGGGCCGTGTCAGACTCCAACCCTGGGATTCTGTGTGGAGAGACATGATAAAATCCAGTCCTTCAAACCAGAGACCTACTGGGTGCTGCAGGCCAAGGTTAACACTGACAAAGACAGATCTCTCCTTTTGGACTGGGACCGAGTAAGAGTGTTTGACCGGGAGATCGCACAGATGTTTTTAAACATGACAAAGCTGGAGAAGGAAGCCCAGGTGGAGGCCACAAGCAGGAAAGAAAAGGCCAAGCAGAGGCCCCTGGCCCTGAACACTGTGGAGATGCTGCGTGTGGCCAGCTCTTCTCTGGGCATGGGGCCGCAGCACGCCATGCAGACGGCTGAGCGGCTCTACACGCAAGGCTACATCAGCTACCCAtggACAGAGACCACCCACTACCCTGAGAACTTTGACCTGAAGGGCTCTCTGCGGCAGCAGGCCAACCACCCCTACTGGGCCGACACGGTGAAGCGGTTGTTAGCAGAAGGTATCAACCGCCCGCGGAAAGGCCATGACGCCGGCGACCATCCCCCCATCACCCCCATGAAGTCTGCCACAGAGGCCGAATTAGGGGGTGACGCGTGGCGGCTCTATGAGTACATCACCAGACACTTCATCGCCACGGTCAGCCATGACTGCAAGTACCTGCAGAGCACCATCTCCTTCAGAATTGGGCCCGAGCTCTTCACCTGCTCCGGGAAGACCGTCCTCTCACCAGGCTTCACGGAGGTCATGCCCTGGCAGAGCGTGCCCCTGGAGGAGAGCCTGCCCACTTGCCAGCGGGGTGATGCCTTCCCTGTGGGCGAGGTGAAGATGCTGGAGAAGCAGACGAACCCACCCGACTACCTGACGGAGGCCGAGCTCATCACGCTCATGGAGAAGCATGGCATCGGCACGGATGCCAGCATCCCTGTGCATATCAACAACATCTGCCAGCGCAACTATGTCACGGTGGAGAGCGGGCGCCGGCTCAAGCCCACCAACCTCGGCATCGTCCTGGTGCACGGCTACTATAAGATTGATGCAGAGCTGGTGCTCCCCACCATCCGCAGTGCAGTGGAGAAGCAGCTGAACCTGATCGCCCAGGGCAAGGCCGACTACCGCCAGGTCCTGGGCCACACCCTGGACGTGTTCAAGAGGAAGTTCCACTACTTTGTCGACTCCATTGCTGGCATGGATGAGTTGATGGAGGTGTCTTTCTCGCCCCTGGCGGCCACAGGCAAGCCCCTCTCACGCTGTGGGAAGTGCCACCGCTTCATGAAGTACATCCAGGCCAAGCCAAGCCGCCTGCACTGCTCCCACTGCGATGAGACCTACACGCTCCCCCAGAACGGCACCATCAAGCTCTACAAGGAGCTCCGCTGCCCTCTGGATGACTTCGAGCTGGTCCTGTGGTCATCAGGCTCTCGGGGCAAGAGCTACCCGCTGTGCCCCTACTGCTACAACCACCCACCCTTCCGAGACATGAAGAAAGGCATGGGCTGCAACGAGTGTACGCACCCCTCCTGCCAGCACTCGCTGAGCATGCTGGGCATCGGCCAGTGCGTGGAATGTGAGAGCGGGGTGCTGGTGCTGGACCCCACCTCGGGCCCCAAGTGGAAGGTGGCCTGCAACAAGTGCAACGTGGTAGCGCACTGCTTCGAGAACGCCCACCGCGTGCGGGTGTCCGCCGACACCTGCAGTGTCTGTGAGGCCGCCTTGCTTGATGTGGACTTCAACAAGGCCAAGTCCCCACTCCCGGGCGATGAGACGCAGCACATGGGCTGCGTCTTTTGTGACCCCGTCTTCCAGGAGCTGGTGGAGCTGAAGCATGCGGCCTCCTGCCACCCCATGCACTAA

**3xFLAG-TOP3B R338W 1-612**

ATGggttcttctgactacaaagaccatgacggtgattataaagatcatgacatcgattacaaggatgacgatgacaagggttcttctAAGACTGTGCTCATGGTTGCTGAAAAGCCGTCCTTGGCACAGTCAATTGCCAAAATCCTCTCTAGAGGGAGCCTGTCCTCACACAAAGGGCTGAACGGGGCCTGCTCAGTCCACGAGTACACTGGGACCTTTGCTGGCCAGCCAGTGCGCTTCAAGATGACGTCTGTCTGTGGTCACGTGATGACCCTGGATTTCCTGGGAAAATACAACAAATGGGACAAAGTGGACCCCGCAGAACTGTTCAGCCAAGCTCCCACGGAGAAGAAAGAAGCTAACCCCAAGCTGAACATGGTGAAGTTCCTGCAGGTGGAGGGCAGAGGCTGCGACTACATCGTGCTGTGGCTGGACTGCGACAAGGAGGGGGAGAACATCTGCTTTGAGGTTCTTGATGCTGTTCTGCCCGTCATGAACAAGGCCCATGGTGGCGAGAAGACCGTGTTCCGGGCCAGGTTTAGCTCCATCACGGACACAGACATCTGTAATGCCATGGCCTGCCTAGGCGAGCCTGACCACAACGAGGCGCTCTCAGTGGATGCTCGCCAGGAGCTGGACCTGCGAATCGGCTGTGCATTCACCAGGTTTCAGACTAAATATTTCCAGGGGAAATACGGTGATTTAGACAGCTCTCTCATCTCCTTTGGGCCGTGTCAGACTCCAACCCTGGGATTCTGTGTGGAGAGACATGATAAAATCCAGTCCTTCAAACCAGAGACCTACTGGGTGCTGCAGGCCAAGGTTAACACTGACAAAGACAGATCTCTCCTTTTGGACTGGGACCGAGTAAGAGTGTTTGACCGGGAGATCGCACAGATGTTTTTAAACATGACAAAGCTGGAGAAGGAAGCCCAGGTGGAGGCCACAAGCAGGAAAGAAAAGGCCAAGCAGAGGCCCCTGGCCCTGAACACTGTGGAGATGCTGCGTGTGGCCAGCTCTTCTCTGGGCATGGGGCCGCAGCACGCCATGCAGACGGCTGAGCGGCTCTACACGCAAGGCTACATCAGCTACCCAtggACAGAGACCACCCACTACCCTGAGAACTTTGACCTGAAGGGCTCTCTGCGGCAGCAGGCCAACCACCCCTACTGGGCCGACACGGTGAAGCGGTTGTTAGCAGAAGGTATCAACCGCCCGCGGAAAGGCCATGACGCCGGCGACCATCCCCCCATCACCCCCATGAAGTCTGCCACAGAGGCCGAATTAGGGGGTGACGCGTGGCGGCTCTATGAGTACATCACCAGACACTTCATCGCCACGGTCAGCCATGACTGCAAGTACCTGCAGAGCACCATCTCCTTCAGAATTGGGCCCGAGCTCTTCACCTGCTCCGGGAAGACCGTCCTCTCACCAGGCTTCACGGAGGTCATGCCCTGGCAGAGCGTGCCCCTGGAGGAGAGCCTGCCCACTTGCCAGCGGGGTGATGCCTTCCCTGTGGGCGAGGTGAAGATGCTGGAGAAGCAGACGAACCCACCCGACTACCTGACGGAGGCCGAGCTCATCACGCTCATGGAGAAGCATGGCATCGGCACGGATGCCAGCATCCCTGTGCATATCAACAACATCTGCCAGCGCAACTATGTCACGGTGGAGAGCGGGCGCCGGCTCAAGCCCACCAACCTCGGCATCGTCCTGGTGCACGGCTACTATAAGATTGATGCAGAGCTGGTGCTCCCCACCATCCGCAGTGCAGTGGAGAAGCAGCTGAACCTGATCGCCCAGGGCAAGGCCGACTACCGCCAGGTCCTGGGCCACACCCTGGACGTGTTCAAGAGGAAGTTCCACTACTTTGTCGACTCCATTGCTGGCATGGATGAGTTGATGGAGGTGTCTTTCTCGTAA

**TOP3B-3xFLAG ZnF (D669A)**

ATGAAGACTGTGCTCATGGTTGCTGAAAAGCCGTCCTTGGCACAGTCAATTGCCAAAATCCTCTCTAGAGGGAGCCTGTCCTCACACAAAGGGCTGAACGGGGCCTGCTCAGTCCACGAGTACACTGGGACCTTTGCTGGCCAGCCAGTGCGCTTCAAGATGACGTCTGTCTGTGGTCACGTGATGACCCTGGATTTCCTGGGAAAATACAACAAATGGGACAAAGTGGACCCCGCAGAACTGTTCAGCCAAGCTCCCACGGAGAAGAAAGAAGCTAACCCCAAGCTGAACATGGTGAAGTTCCTGCAGGTGGAGGGCAGAGGCTGCGACTACATCGTGCTGTGGCTGGACTGCGACAAGGAGGGGGAGAACATCTGCTTTGAGGTTCTTGATGCTGTTCTGCCCGTCATGAACAAGGCCCATGGTGGCGAGAAGACCGTGTTCCGGGCCAGGTTTAGCTCCATCACGGACACAGACATCTGTAATGCCATGGCCTGCCTAGGCGAGCCTGACCACAACGAGGCGCTCTCAGTGGATGCTCGCCAGGAGCTGGACCTGCGAATCGGCTGTGCATTCACCAGGTTTCAGACTAAATATTTCCAGGGGAAATACGGTGATTTAGACAGCTCTCTCATCTCCTTTGGGCCGTGTCAGACTCCAACCCTGGGATTCTGTGTGGAGAGACATGATAAAATCCAGTCCTTCAAACCAGAGACCTACTGGGTGCTGCAGGCCAAGGTTAACACTGACAAAGACAGATCTCTCCTTTTGGACTGGGACCGAGTAAGAGTGTTTGACCGGGAGATCGCACAGATGTTTTTAAACATGACAAAGCTGGAGAAGGAAGCCCAGGTGGAGGCCACAAGCAGGAAAGAAAAGGCCAAGCAGAGGCCCCTGGCCCTGAACACTGTGGAGATGCTGCGTGTGGCCAGCTCTTCTCTGGGCATGGGGCCGCAGCACGCCATGCAGACGGCTGAGCGGCTCTACACGCAAGGCTACATCAGCTACCCACGGACAGAGACCACCCACTACCCTGAGAACTTTGACCTGAAGGGCTCTCTGCGGCAGCAGGCCAACCACCCCTACTGGGCCGACACGGTGAAGCGGTTGTTAGCAGAAGGTATCAACCGCCCGCGGAAAGGCCATGACGCCGGCGACCATCCCCCCATCACCCCCATGAAGTCTGCCACAGAGGCCGAATTAGGGGGTGACGCGTGGCGGCTCTATGAGTACATCACCAGACACTTCATCGCCACGGTCAGCCATGACTGCAAGTACCTGCAGAGCACCATCTCCTTCAGAATTGGGCCCGAGCTCTTCACCTGCTCCGGGAAGACCGTCCTCTCACCAGGCTTCACGGAGGTCATGCCCTGGCAGAGCGTGCCCCTGGAGGAGAGCCTGCCCACTTGCCAGCGGGGTGATGCCTTCCCTGTGGGCGAGGTGAAGATGCTGGAGAAGCAGACGAACCCACCCGACTACCTGACGGAGGCCGAGCTCATCACGCTCATGGAGAAGCATGGCATCGGCACGGATGCCAGCATCCCTGTGCATATCAACAACATCTGCCAGCGCAACTATGTCACGGTGGAGAGCGGGCGCCGGCTCAAGCCCACCAACCTCGGCATCGTCCTGGTGCACGGCTACTATAAGATTGATGCAGAGCTGGTGCTCCCCACCATCCGCAGTGCAGTGGAGAAGCAGCTGAACCTGATCGCCCAGGGCAAGGCCGACTACCGCCAGGTCCTGGGCCACACCCTGGACGTGTTCAAGAGGAAGTTCCACTACTTTGTCGACTCCATTGCTGGCATGGATGAGTTGATGGAGGTGTCTTTCTCGCCCCTGGCGGCCACAGGCAAGCCCCTCTCACGCTGTGGGAAGTGCCACCGCTTCATGAAGTACATCCAGGCCAAGCCAAGCCGCCTGCACTGCTCCCACTGCGATGAGACCTACACGCTCCCCCAGAACGGCACCATCAAGCTCTACAAGGAGCTCCGCTGCCCTCTGgccGACTTCGAGCTGGTCCTGTGGTCATCAGGCTCTCGGGGCAAGAGCTACCCGCTGTGCCCCTACTGCTACAACCACCCACCCTTCCGAGACATGAAGAAAGGCATGGGCTGCAACGAGTGTACGCACCCCTCCTGCCAGCACTCGCTGAGCATGCTGGGCATCGGCCAGTGCGTGGAATGTGAGAGCGGGGTGCTGGTGCTGGACCCCACCTCGGGCCCCAAGTGGAAGGTGGCCTGCAACAAGTGCAACGTGGTAGCGCACTGCTTCGAGAACGCCCACCGCGTGCGGGTGTCCGCCGACACCTGCAGTGTCTGTGAGGCCGCCTTGCTTGATGTGGACTTCAACAAGGCCAAGTCCCCACTCCCGGGCGATGAGACGCAGCACATGGGCTGCGTCTTTTGTGACCCCGTCTTCCAGGAGCTGGTGGAGCTGAAGCATGCGGCCTCCTGCCACCCCATGCACCGCGGTGGACCAGGGAGAAGGCAGGGTCGAGGGCGGGGCCGGGCCAGGAGGCCCCCTGGGAAGCCCAACCCCAGACGGCCCAAGGACAAGATGTCAGCCCTGGCCGCCTACTTTGTAAGCGGACCGACGCGTACGCGGCCGCTCGAGCAGAAACTCATCTCAGAAGAGGATCTGGCAGCAAATGATATCCTGgactacaaagaccatgacggtgattataaagatcatgacatcgattacaaggatgacgatgacaagGTTTAA

**TOP3B-3xFLAG ZnF (C688A/C691A)**

ATGAAGACTGTGCTCATGGTTGCTGAAAAGCCGTCCTTGGCACAGTCAATTGCCAAAATCCTCTCTAGAGGGAGCCTGTCCTCACACAAAGGGCTGAACGGGGCCTGCTCAGTCCACGAGTACACTGGGACCTTTGCTGGCCAGCCAGTGCGCTTCAAGATGACGTCTGTCTGTGGTCACGTGATGACCCTGGATTTCCTGGGAAAATACAACAAATGGGACAAAGTGGACCCCGCAGAACTGTTCAGCCAAGCTCCCACGGAGAAGAAAGAAGCTAACCCCAAGCTGAACATGGTGAAGTTCCTGCAGGTGGAGGGCAGAGGCTGCGACTACATCGTGCTGTGGCTGGACTGCGACAAGGAGGGGGAGAACATCTGCTTTGAGGTTCTTGATGCTGTTCTGCCCGTCATGAACAAGGCCCATGGTGGCGAGAAGACCGTGTTCCGGGCCAGGTTTAGCTCCATCACGGACACAGACATCTGTAATGCCATGGCCTGCCTAGGCGAGCCTGACCACAACGAGGCGCTCTCAGTGGATGCTCGCCAGGAGCTGGACCTGCGAATCGGCTGTGCATTCACCAGGTTTCAGACTAAATATTTCCAGGGGAAATACGGTGATTTAGACAGCTCTCTCATCTCCTTTGGGCCGTGTCAGACTCCAACCCTGGGATTCTGTGTGGAGAGACATGATAAAATCCAGTCCTTCAAACCAGAGACCTACTGGGTGCTGCAGGCCAAGGTTAACACTGACAAAGACAGATCTCTCCTTTTGGACTGGGACCGAGTAAGAGTGTTTGACCGGGAGATCGCACAGATGTTTTTAAACATGACAAAGCTGGAGAAGGAAGCCCAGGTGGAGGCCACAAGCAGGAAAGAAAAGGCCAAGCAGAGGCCCCTGGCCCTGAACACTGTGGAGATGCTGCGTGTGGCCAGCTCTTCTCTGGGCATGGGGCCGCAGCACGCCATGCAGACGGCTGAGCGGCTCTACACGCAAGGCTACATCAGCTACCCACGGACAGAGACCACCCACTACCCTGAGAACTTTGACCTGAAGGGCTCTCTGCGGCAGCAGGCCAACCACCCCTACTGGGCCGACACGGTGAAGCGGTTGTTAGCAGAAGGTATCAACCGCCCGCGGAAAGGCCATGACGCCGGCGACCATCCCCCCATCACCCCCATGAAGTCTGCCACAGAGGCCGAATTAGGGGGTGACGCGTGGCGGCTCTATGAGTACATCACCAGACACTTCATCGCCACGGTCAGCCATGACTGCAAGTACCTGCAGAGCACCATCTCCTTCAGAATTGGGCCCGAGCTCTTCACCTGCTCCGGGAAGACCGTCCTCTCACCAGGCTTCACGGAGGTCATGCCCTGGCAGAGCGTGCCCCTGGAGGAGAGCCTGCCCACTTGCCAGCGGGGTGATGCCTTCCCTGTGGGCGAGGTGAAGATGCTGGAGAAGCAGACGAACCCACCCGACTACCTGACGGAGGCCGAGCTCATCACGCTCATGGAGAAGCATGGCATCGGCACGGATGCCAGCATCCCTGTGCATATCAACAACATCTGCCAGCGCAACTATGTCACGGTGGAGAGCGGGCGCCGGCTCAAGCCCACCAACCTCGGCATCGTCCTGGTGCACGGCTACTATAAGATTGATGCAGAGCTGGTGCTCCCCACCATCCGCAGTGCAGTGGAGAAGCAGCTGAACCTGATCGCCCAGGGCAAGGCCGACTACCGCCAGGTCCTGGGCCACACCCTGGACGTGTTCAAGAGGAAGTTCCACTACTTTGTCGACTCCATTGCTGGCATGGATGAGTTGATGGAGGTGTCTTTCTCGCCCCTGGCGGCCACAGGCAAGCCCCTCTCACGCTGTGGGAAGTGCCACCGCTTCATGAAGTACATCCAGGCCAAGCCAAGCCGCCTGCACTGCTCCCACTGCGATGAGACCTACACGCTCCCCCAGAACGGCACCATCAAGCTCTACAAGGAGCTCCGCTGCCCTCTGGATGACTTCGAGCTGGTCCTGTGGTCATCAGGCTCTCGGGGCAAGAGCTACCCGCTGgccCCCTACgccTACAACCACCCACCCTTCCGAGACATGAAGAAAGGCATGGGCTGCAACGAGTGTACGCACCCCTCCTGCCAGCACTCGCTGAGCATGCTGGGCATCGGCCAGTGCGTGGAATGTGAGAGCGGGGTGCTGGTGCTGGACCCCACCTCGGGCCCCAAGTGGAAGGTGGCCTGCAACAAGTGCAACGTGGTAGCGCACTGCTTCGAGAACGCCCACCGCGTGCGGGTGTCCGCCGACACCTGCAGTGTCTGTGAGGCCGCCTTGCTTGATGTGGACTTCAACAAGGCCAAGTCCCCACTCCCGGGCGATGAGACGCAGCACATGGGCTGCGTCTTTTGTGACCCCGTCTTCCAGGAGCTGGTGGAGCTGAAGCATGCGGCCTCCTGCCACCCCATGCACCGCGGTGGACCAGGGAGAAGGCAGGGTCGAGGGCGGGGCCGGGCCAGGAGGCCCCCTGGGAAGCCCAACCCCAGACGGCCCAAGGACAAGATGTCAGCCCTGGCCGCCTACTTTGTAAGCGGACCGACGCGTACGCGGCCGCTCGAGCAGAAACTCATCTCAGAAGAGGATCTGGCAGCAAATGATATCCTGgactacaaagaccatgacggtgattataaagatcatgacatcgattacaaggatgacgatgacaagGTTTAA

**TOP3B-3xFLAG ZnF (C706A/C709A)**

ATGAAGACTGTGCTCATGGTTGCTGAAAAGCCGTCCTTGGCACAGTCAATTGCCAAAATCCTCTCTAGAGGGAGCCTGTCCTCACACAAAGGGCTGAACGGGGCCTGCTCAGTCCACGAGTACACTGGGACCTTTGCTGGCCAGCCAGTGCGCTTCAAGATGACGTCTGTCTGTGGTCACGTGATGACCCTGGATTTCCTGGGAAAATACAACAAATGGGACAAAGTGGACCCCGCAGAACTGTTCAGCCAAGCTCCCACGGAGAAGAAAGAAGCTAACCCCAAGCTGAACATGGTGAAGTTCCTGCAGGTGGAGGGCAGAGGCTGCGACTACATCGTGCTGTGGCTGGACTGCGACAAGGAGGGGGAGAACATCTGCTTTGAGGTTCTTGATGCTGTTCTGCCCGTCATGAACAAGGCCCATGGTGGCGAGAAGACCGTGTTCCGGGCCAGGTTTAGCTCCATCACGGACACAGACATCTGTAATGCCATGGCCTGCCTAGGCGAGCCTGACCACAACGAGGCGCTCTCAGTGGATGCTCGCCAGGAGCTGGACCTGCGAATCGGCTGTGCATTCACCAGGTTTCAGACTAAATATTTCCAGGGGAAATACGGTGATTTAGACAGCTCTCTCATCTCCTTTGGGCCGTGTCAGACTCCAACCCTGGGATTCTGTGTGGAGAGACATGATAAAATCCAGTCCTTCAAACCAGAGACCTACTGGGTGCTGCAGGCCAAGGTTAACACTGACAAAGACAGATCTCTCCTTTTGGACTGGGACCGAGTAAGAGTGTTTGACCGGGAGATCGCACAGATGTTTTTAAACATGACAAAGCTGGAGAAGGAAGCCCAGGTGGAGGCCACAAGCAGGAAAGAAAAGGCCAAGCAGAGGCCCCTGGCCCTGAACACTGTGGAGATGCTGCGTGTGGCCAGCTCTTCTCTGGGCATGGGGCCGCAGCACGCCATGCAGACGGCTGAGCGGCTCTACACGCAAGGCTACATCAGCTACCCACGGACAGAGACCACCCACTACCCTGAGAACTTTGACCTGAAGGGCTCTCTGCGGCAGCAGGCCAACCACCCCTACTGGGCCGACACGGTGAAGCGGTTGTTAGCAGAAGGTATCAACCGCCCGCGGAAAGGCCATGACGCCGGCGACCATCCCCCCATCACCCCCATGAAGTCTGCCACAGAGGCCGAATTAGGGGGTGACGCGTGGCGGCTCTATGAGTACATCACCAGACACTTCATCGCCACGGTCAGCCATGACTGCAAGTACCTGCAGAGCACCATCTCCTTCAGAATTGGGCCCGAGCTCTTCACCTGCTCCGGGAAGACCGTCCTCTCACCAGGCTTCACGGAGGTCATGCCCTGGCAGAGCGTGCCCCTGGAGGAGAGCCTGCCCACTTGCCAGCGGGGTGATGCCTTCCCTGTGGGCGAGGTGAAGATGCTGGAGAAGCAGACGAACCCACCCGACTACCTGACGGAGGCCGAGCTCATCACGCTCATGGAGAAGCATGGCATCGGCACGGATGCCAGCATCCCTGTGCATATCAACAACATCTGCCAGCGCAACTATGTCACGGTGGAGAGCGGGCGCCGGCTCAAGCCCACCAACCTCGGCATCGTCCTGGTGCACGGCTACTATAAGATTGATGCAGAGCTGGTGCTCCCCACCATCCGCAGTGCAGTGGAGAAGCAGCTGAACCTGATCGCCCAGGGCAAGGCCGACTACCGCCAGGTCCTGGGCCACACCCTGGACGTGTTCAAGAGGAAGTTCCACTACTTTGTCGACTCCATTGCTGGCATGGATGAGTTGATGGAGGTGTCTTTCTCGCCCCTGGCGGCCACAGGCAAGCCCCTCTCACGCTGTGGGAAGTGCCACCGCTTCATGAAGTACATCCAGGCCAAGCCAAGCCGCCTGCACTGCTCCCACTGCGATGAGACCTACACGCTCCCCCAGAACGGCACCATCAAGCTCTACAAGGAGCTCCGCTGCCCTCTGGATGACTTCGAGCTGGTCCTGTGGTCATCAGGCTCTCGGGGCAAGAGCTACCCGCTGTGCCCCTACTGCTACAACCACCCACCCTTCCGAGACATGAAGAAAGGCATGGGCgccAACGAGgccACGCACCCCTCCTGCCAGCACTCGCTGAGCATGCTGGGCATCGGCCAGTGCGTGGAATGTGAGAGCGGGGTGCTGGTGCTGGACCCCACCTCGGGCCCCAAGTGGAAGGTGGCCTGCAACAAGTGCAACGTGGTAGCGCACTGCTTCGAGAACGCCCACCGCGTGCGGGTGTCCGCCGACACCTGCAGTGTCTGTGAGGCCGCCTTGCTTGATGTGGACTTCAACAAGGCCAAGTCCCCACTCCCGGGCGATGAGACGCAGCACATGGGCTGCGTCTTTTGTGACCCCGTCTTCCAGGAGCTGGTGGAGCTGAAGCATGCGGCCTCCTGCCACCCCATGCACCGCGGTGGACCAGGGAGAAGGCAGGGTCGAGGGCGGGGCCGGGCCAGGAGGCCCCCTGGGAAGCCCAACCCCAGACGGCCCAAGGACAAGATGTCAGCCCTGGCCGCCTACTTTGTAAGCGGACCGACGCGTACGCGGCCGCTCGAGCAGAAACTCATCTCAGAAGAGGATCTGGCAGCAAATGATATCCTGgactacaaagaccatgacggtgattataaagatcatgacatcgattacaaggatgacgatgacaagGTTTAA

**mEGFP (used in pcDNA3.1(+))**

atggtgagcaagggcgaggagctgttcaccggggtggtgcccatcctggtcgagctggacggcgacgtaaacggccacaagttcagcgtgtccggcgagggcgagggcgatgccacctacggcaagctgaccctgaagttcatctgcaccaccggcaagctgcccgtgccctggcccaccctcgtgaccaccctgacctacggcgtgcagtgcttcagccgctaccccgaccacatgaagcagcacgacttcttcaagtccgccatgcccgaaggctacgtccaggagcgcaccatcttcttcaaggacgacggcaactacaagacccgcgccgaggtgaagttcgagggcgacaccctggtgaaccgcatcgagctgaagggcatcgacttcaaggaggacggcaacatcctggggcacaagctggagtacaactacaacagccacaacgtctatatcatggccgacaagcagaagaacggcatcaaggtgaacttcaagatccgccacaacatcgaggacggcagcgtgcagctcgccgaccactaccagcagaacacccccatcggcgacggccccgtgctgctgcccgacaaccactacctgagcacccagtccaagctgagcaaagaccccaacgagaagcgcgatcacatggtcctgctggagttcgtgaccgccgccgggatcactctcggcatggacgagctgtacaagtaa

**3xV5-Ubiquitn (used in pcDNA3.1(+))**

atggggaagcctatacccaatcctctcctggggttggacagcaccggcagtggtagcggtggcaagcccatccccaaccccctgctgggcctggactccaccggctccggcagtggtggaaaacctattcccaacccactgctgggactggattctacaagcggatccatgcagatcttcgtgaaaacccttaccggcaagaccatcacccttgaggtggagcccagtgacaccatcgaaaatgtgaaggccaagatccaggataaggaaggcattccccccgaccagcagaggctcatctttgcaggcaagcagctggaagatggccgtactctttctgactacaacatccagaaggagtcgaccctgcacctggtcctgcgtctgagaggtggttag

**3xV5-Ubiquitn K6R, K27R, K29R, K33R, K63R (used in pcDNA3.1(+))**

atggggaagcctatacccaatcctctcctggggttggacagcaccggcagtggtagcggtggcaagcccatccccaaccccctgctgggcctggactccaccggctccggcagtggtggaaaacctattcccaacccactgctgggactggattctacaagcggatccatgcagatcttcgtgCGCacccttaccggcaagaccatcacccttgaggtggagcccagtgacaccatcgaaaatgtgCGCgccCGCatccaggatCGCgaaggcattccccccgaccagcagaggctcatctttgcaggcaagcagctggaagatggccgtactctttctgactacaacatccagCGCgagtcgaccctgcacctggtcctgcgtctgagaggtggttag

**3xV5-Ubiquitn K11R, K48R (used in pcDNA3.1(+))**

atggggaagcctatacccaatcctctcctggggttggacagcaccggcagtggtagcggtggcaagcccatccccaaccccctgctgggcctggactccaccggctccggcagtggtggaaaacctattcccaacccactgctgggactggattctacaagcggatccatgcagatcttcgtgaaaacccttaccggcCGCaccatcacccttgaggtggagcccagtgacaccatcgaaaatgtgaaggccaagatccaggataaggaaggcattccccccgaccagcagaggctcatctttgcaggcCGCcagctggaagatggccgtactctttctgactacaacatccagaaggagtcgaccctgcacctggtcctgcgtctgagaggtggttag

**ORFs in plasmids used in longitudinal fluorescence microscopy and to generate dox-inducible stable cell lines**

**3xFLAG-mEGFP-NES-TOP3B WT (used in pGW1 and pcDNA5/FRT/TO)**

atgggaagcagcgactacaaagaccatgacggtgattataaagatcatgacatcgattacaaggatgacgatgacaagggttctggtgtgagcaagggcgaggagctgttcaccggggtggtgcccatcctggtcgagctggacggcgacgtaaacggccacaagttcagcgtgtccggcgagggcgagggcgatgccacctacggcaagctgaccctgaagttcatctgcaccaccggcaagctgcccgtgccctggcccaccctcgtgaccaccctgacctacggcgtgcagtgcttcagccgctaccccgaccacatgaagcagcacgacttcttcaagtccgccatgcccgaaggctacgtccaggagcgcaccatcttcttcaaggacgacggcaactacaagacccgcgccgaggtgaagttcgagggcgacaccctggtgaaccgcatcgagctgaagggcatcgacttcaaggaggacggcaacatcctggggcacaagctggagtacaactacaacagccacaacgtctatatcatggccgacaagcagaagaacggcatcaaggtgaacttcaagatccgccacaacatcgaggacggcagcgtgcagctcgccgaccactaccagcagaacacccccatcggcgacggccccgtgctgctgcccgacaaccactacctgagcacccagtccaagctgagcaaagaccccaacgagaagcgcgatcacatggtcctgctggagttcgtgaccgccgccgggatcactctcggcatggacgagctgtacaagggttctggtctgcagctgcctcccctggagcgcctgaccctggacggttcttctAAGACTGTGCTCATGGTTGCTGAAAAGCCGTCCTTGGCACAGTCAATTGCCAAAATCCTCTCTAGAGGGAGCCTGTCCTCACACAAAGGGCTGAACGGGGCCTGCTCAGTCCACGAGTACACTGGGACCTTTGCTGGCCAGCCAGTGCGCTTCAAGATGACGTCTGTCTGTGGTCACGTGATGACCCTGGATTTCCTGGGAAAATACAACAAATGGGACAAAGTGGACCCCGCAGAACTGTTCAGCCAAGCTCCCACGGAGAAGAAAGAAGCTAACCCCAAGCTGAACATGGTGAAGTTCCTGCAGGTGGAGGGCAGAGGCTGCGACTACATCGTGCTGTGGCTGGACTGCGACAAGGAGGGGGAGAACATCTGCTTTGAGGTTCTTGATGCTGTTCTGCCCGTCATGAACAAGGCCCATGGTGGCGAGAAGACCGTGTTCCGGGCCAGGTTTAGCTCCATCACGGACACAGACATCTGTAATGCCATGGCCTGCCTAGGCGAGCCTGACCACAACGAGGCGCTCTCAGTGGATGCTCGCCAGGAGCTGGACCTGCGAATCGGCTGTGCATTCACCAGGTTTCAGACTAAATATTTCCAGGGGAAATACGGTGATTTAGACAGCTCTCTCATCTCCTTTGGGCCGTGTCAGACTCCAACCCTGGGATTCTGTGTGGAGAGACATGATAAAATCCAGTCCTTCAAACCAGAGACCTACTGGGTGCTGCAGGCCAAGGTTAACACTGACAAAGACAGATCTCTCCTTTTGGACTGGGACCGAGTAAGAGTGTTTGACCGGGAGATCGCACAGATGTTTTTAAACATGACAAAGCTGGAGAAGGAAGCCCAGGTGGAGGCCACAAGCAGGAAAGAAAAGGCCAAGCAGAGGCCCCTGGCCCTGAACACTGTGGAGATGCTGCGTGTGGCCAGCTCTTCTCTGGGCATGGGGCCGCAGCACGCCATGCAGACGGCTGAGCGGCTCTACACGCAAGGCTACATCAGCTACCCACGGACAGAGACCACCCACTACCCTGAGAACTTTGACCTGAAGGGCTCTCTGCGGCAGCAGGCCAACCACCCCTACTGGGCCGACACGGTGAAGCGGTTGTTAGCAGAAGGTATCAACCGCCCGCGGAAAGGCCATGACGCCGGCGACCATCCCCCCATCACCCCCATGAAGTCTGCCACAGAGGCCGAATTAGGGGGTGACGCGTGGCGGCTCTATGAGTACATCACCAGACACTTCATCGCCACGGTCAGCCATGACTGCAAGTACCTGCAGAGCACCATCTCCTTCAGAATTGGGCCCGAGCTCTTCACCTGCTCCGGGAAGACCGTCCTCTCACCAGGCTTCACGGAGGTCATGCCCTGGCAGAGCGTGCCCCTGGAGGAGAGCCTGCCCACTTGCCAGCGGGGTGATGCCTTCCCTGTGGGCGAGGTGAAGATGCTGGAGAAGCAGACGAACCCACCCGACTACCTGACGGAGGCCGAGCTCATCACGCTCATGGAGAAGCATGGCATCGGCACGGATGCCAGCATCCCTGTGCATATCAACAACATCTGCCAGCGCAACTATGTCACGGTGGAGAGCGGGCGCCGGCTCAAGCCCACCAACCTCGGCATCGTCCTGGTGCACGGCTACTATAAGATTGATGCAGAGCTGGTGCTCCCCACCATCCGCAGTGCAGTGGAGAAGCAGCTGAACCTGATCGCCCAGGGCAAGGCCGACTACCGCCAGGTCCTGGGCCACACCCTGGACGTGTTCAAGAGGAAGTTCCACTACTTTGTCGACTCCATTGCTGGCATGGATGAGTTGATGGAGGTGTCTTTCTCGCCCCTGGCGGCCACAGGCAAGCCCCTCTCACGCTGTGGGAAGTGCCACCGCTTCATGAAGTACATCCAGGCCAAGCCAAGCCGCCTGCACTGCTCCCACTGCGATGAGACCTACACGCTCCCCCAGAACGGCACCATCAAGCTCTACAAGGAGCTCCGCTGCCCTCTGGATGACTTCGAGCTGGTCCTGTGGTCATCAGGCTCTCGGGGCAAGAGCTACCCGCTGTGCCCCTACTGCTACAACCACCCACCCTTCCGAGACATGAAGAAAGGCATGGGCTGCAACGAGTGTACGCACCCCTCCTGCCAGCACTCGCTGAGCATGCTGGGCATCGGCCAGTGCGTGGAATGTGAGAGCGGGGTGCTGGTGCTGGACCCCACCTCGGGCCCCAAGTGGAAGGTGGCCTGCAACAAGTGCAACGTGGTAGCGCACTGCTTCGAGAACGCCCACCGCGTGCGGGTGTCCGCCGACACCTGCAGTGTCTGTGAGGCCGCCTTGCTTGATGTGGACTTCAACAAGGCCAAGTCCCCACTCCCGGGCGATGAGACGCAGCACATGGGCTGCGTCTTTTGTGACCCCGTCTTCCAGGAGCTGGTGGAGCTGAAGCATGCGGCCTCCTGCCACCCCATGCACCGCGGTGGACCAGGGAGAAGGCAGGGTCGAGGGCGGGGCCGGGCCAGGAGGCCCCCTGGGAAGCCCAACCCCAGACGGCCCAAGGACAAGATGTCAGCCCTGGCCGCCTACTTTGTATAA

**3xFLAG-mEGFP-NES-TOP3B Y336F (used in pGW1 and pcDNA5/FRT/TO)**

atgggaagcagcgactacaaagaccatgacggtgattataaagatcatgacatcgattacaaggatgacgatgacaagggttctggtgtgagcaagggcgaggagctgttcaccggggtggtgcccatcctggtcgagctggacggcgacgtaaacggccacaagttcagcgtgtccggcgagggcgagggcgatgccacctacggcaagctgaccctgaagttcatctgcaccaccggcaagctgcccgtgccctggcccaccctcgtgaccaccctgacctacggcgtgcagtgcttcagccgctaccccgaccacatgaagcagcacgacttcttcaagtccgccatgcccgaaggctacgtccaggagcgcaccatcttcttcaaggacgacggcaactacaagacccgcgccgaggtgaagttcgagggcgacaccctggtgaaccgcatcgagctgaagggcatcgacttcaaggaggacggcaacatcctggggcacaagctggagtacaactacaacagccacaacgtctatatcatggccgacaagcagaagaacggcatcaaggtgaacttcaagatccgccacaacatcgaggacggcagcgtgcagctcgccgaccactaccagcagaacacccccatcggcgacggccccgtgctgctgcccgacaaccactacctgagcacccagtccaagctgagcaaagaccccaacgagaagcgcgatcacatggtcctgctggagttcgtgaccgccgccgggatcactctcggcatggacgagctgtacaagggttctggtctgcagctgcctcccctggagcgcctgaccctggacggttcttctAAGACTGTGCTCATGGTTGCTGAAAAGCCGTCCTTGGCACAGTCAATTGCCAAAATCCTCTCTAGAGGGAGCCTGTCCTCACACAAAGGGCTGAACGGGGCCTGCTCAGTCCACGAGTACACTGGGACCTTTGCTGGCCAGCCAGTGCGCTTCAAGATGACGTCTGTCTGTGGTCACGTGATGACCCTGGATTTCCTGGGAAAATACAACAAATGGGACAAAGTGGACCCCGCAGAACTGTTCAGCCAAGCTCCCACGGAGAAGAAAGAAGCTAACCCCAAGCTGAACATGGTGAAGTTCCTGCAGGTGGAGGGCAGAGGCTGCGACTACATCGTGCTGTGGCTGGACTGCGACAAGGAGGGGGAGAACATCTGCTTTGAGGTTCTTGATGCTGTTCTGCCCGTCATGAACAAGGCCCATGGTGGCGAGAAGACCGTGTTCCGGGCCAGGTTTAGCTCCATCACGGACACAGACATCTGTAATGCCATGGCCTGCCTAGGCGAGCCTGACCACAACGAGGCGCTCTCAGTGGATGCTCGCCAGGAGCTGGACCTGCGAATCGGCTGTGCATTCACCAGGTTTCAGACTAAATATTTCCAGGGGAAATACGGTGATTTAGACAGCTCTCTCATCTCCTTTGGGCCGTGTCAGACTCCAACCCTGGGATTCTGTGTGGAGAGACATGATAAAATCCAGTCCTTCAAACCAGAGACCTACTGGGTGCTGCAGGCCAAGGTTAACACTGACAAAGACAGATCTCTCCTTTTGGACTGGGACCGAGTAAGAGTGTTTGACCGGGAGATCGCACAGATGTTTTTAAACATGACAAAGCTGGAGAAGGAAGCCCAGGTGGAGGCCACAAGCAGGAAAGAAAAGGCCAAGCAGAGGCCCCTGGCCCTGAACACTGTGGAGATGCTGCGTGTGGCCAGCTCTTCTCTGGGCATGGGGCCGCAGCACGCCATGCAGACGGCTGAGCGGCTCTACACGCAAGGCTACATCAGCtttCCACGGACAGAGACCACCCACTACCCTGAGAACTTTGACCTGAAGGGCTCTCTGCGGCAGCAGGCCAACCACCCCTACTGGGCCGACACGGTGAAGCGGTTGTTAGCAGAAGGTATCAACCGCCCGCGGAAAGGCCATGACGCCGGCGACCATCCCCCCATCACCCCCATGAAGTCTGCCACAGAGGCCGAATTAGGGGGTGACGCGTGGCGGCTCTATGAGTACATCACCAGACACTTCATCGCCACGGTCAGCCATGACTGCAAGTACCTGCAGAGCACCATCTCCTTCAGAATTGGGCCCGAGCTCTTCACCTGCTCCGGGAAGACCGTCCTCTCACCAGGCTTCACGGAGGTCATGCCCTGGCAGAGCGTGCCCCTGGAGGAGAGCCTGCCCACTTGCCAGCGGGGTGATGCCTTCCCTGTGGGCGAGGTGAAGATGCTGGAGAAGCAGACGAACCCACCCGACTACCTGACGGAGGCCGAGCTCATCACGCTCATGGAGAAGCATGGCATCGGCACGGATGCCAGCATCCCTGTGCATATCAACAACATCTGCCAGCGCAACTATGTCACGGTGGAGAGCGGGCGCCGGCTCAAGCCCACCAACCTCGGCATCGTCCTGGTGCACGGCTACTATAAGATTGATGCAGAGCTGGTGCTCCCCACCATCCGCAGTGCAGTGGAGAAGCAGCTGAACCTGATCGCCCAGGGCAAGGCCGACTACCGCCAGGTCCTGGGCCACACCCTGGACGTGTTCAAGAGGAAGTTCCACTACTTTGTCGACTCCATTGCTGGCATGGATGAGTTGATGGAGGTGTCTTTCTCGCCCCTGGCGGCCACAGGCAAGCCCCTCTCACGCTGTGGGAAGTGCCACCGCTTCATGAAGTACATCCAGGCCAAGCCAAGCCGCCTGCACTGCTCCCACTGCGATGAGACCTACACGCTCCCCCAGAACGGCACCATCAAGCTCTACAAGGAGCTCCGCTGCCCTCTGGATGACTTCGAGCTGGTCCTGTGGTCATCAGGCTCTCGGGGCAAGAGCTACCCGCTGTGCCCCTACTGCTACAACCACCCACCCTTCCGAGACATGAAGAAAGGCATGGGCTGCAACGAGTGTACGCACCCCTCCTGCCAGCACTCGCTGAGCATGCTGGGCATCGGCCAGTGCGTGGAATGTGAGAGCGGGGTGCTGGTGCTGGACCCCACCTCGGGCCCCAAGTGGAAGGTGGCCTGCAACAAGTGCAACGTGGTAGCGCACTGCTTCGAGAACGCCCACCGCGTGCGGGTGTCCGCCGACACCTGCAGTGTCTGTGAGGCCGCCTTGCTTGATGTGGACTTCAACAAGGCCAAGTCCCCACTCCCGGGCGATGAGACGCAGCACATGGGCTGCGTCTTTTGTGACCCCGTCTTCCAGGAGCTGGTGGAGCTGAAGCATGCGGCCTCCTGCCACCCCATGCACCGCGGTGGACCAGGGAGAAGGCAGGGTCGAGGGCGGGGCCGGGCCAGGAGGCCCCCTGGGAAGCCCAACCCCAGACGGCCCAAGGACAAGATGTCAGCCCTGGCCGCCTACTTTGTATAA

**3xFLAG-mEGFP-NES-TOP3B R338W (used in pGW1 and pcDNA5/FRT/TO)**

atgggaagcagcgactacaaagaccatgacggtgattataaagatcatgacatcgattacaaggatgacgatgacaagggttctggtgtgagcaagggcgaggagctgttcaccggggtggtgcccatcctggtcgagctggacggcgacgtaaacggccacaagttcagcgtgtccggcgagggcgagggcgatgccacctacggcaagctgaccctgaagttcatctgcaccaccggcaagctgcccgtgccctggcccaccctcgtgaccaccctgacctacggcgtgcagtgcttcagccgctaccccgaccacatgaagcagcacgacttcttcaagtccgccatgcccgaaggctacgtccaggagcgcaccatcttcttcaaggacgacggcaactacaagacccgcgccgaggtgaagttcgagggcgacaccctggtgaaccgcatcgagctgaagggcatcgacttcaaggaggacggcaacatcctggggcacaagctggagtacaactacaacagccacaacgtctatatcatggccgacaagcagaagaacggcatcaaggtgaacttcaagatccgccacaacatcgaggacggcagcgtgcagctcgccgaccactaccagcagaacacccccatcggcgacggccccgtgctgctgcccgacaaccactacctgagcacccagtccaagctgagcaaagaccccaacgagaagcgcgatcacatggtcctgctggagttcgtgaccgccgccgggatcactctcggcatggacgagctgtacaagggttctggtctgcagctgcctcccctggagcgcctgaccctggacggttcttctAAGACTGTGCTCATGGTTGCTGAAAAGCCGTCCTTGGCACAGTCAATTGCCAAAATCCTCTCTAGAGGGAGCCTGTCCTCACACAAAGGGCTGAACGGGGCCTGCTCAGTCCACGAGTACACTGGGACCTTTGCTGGCCAGCCAGTGCGCTTCAAGATGACGTCTGTCTGTGGTCACGTGATGACCCTGGATTTCCTGGGAAAATACAACAAATGGGACAAAGTGGACCCCGCAGAACTGTTCAGCCAAGCTCCCACGGAGAAGAAAGAAGCTAACCCCAAGCTGAACATGGTGAAGTTCCTGCAGGTGGAGGGCAGAGGCTGCGACTACATCGTGCTGTGGCTGGACTGCGACAAGGAGGGGGAGAACATCTGCTTTGAGGTTCTTGATGCTGTTCTGCCCGTCATGAACAAGGCCCATGGTGGCGAGAAGACCGTGTTCCGGGCCAGGTTTAGCTCCATCACGGACACAGACATCTGTAATGCCATGGCCTGCCTAGGCGAGCCTGACCACAACGAGGCGCTCTCAGTGGATGCTCGCCAGGAGCTGGACCTGCGAATCGGCTGTGCATTCACCAGGTTTCAGACTAAATATTTCCAGGGGAAATACGGTGATTTAGACAGCTCTCTCATCTCCTTTGGGCCGTGTCAGACTCCAACCCTGGGATTCTGTGTGGAGAGACATGATAAAATCCAGTCCTTCAAACCAGAGACCTACTGGGTGCTGCAGGCCAAGGTTAACACTGACAAAGACAGATCTCTCCTTTTGGACTGGGACCGAGTAAGAGTGTTTGACCGGGAGATCGCACAGATGTTTTTAAACATGACAAAGCTGGAGAAGGAAGCCCAGGTGGAGGCCACAAGCAGGAAAGAAAAGGCCAAGCAGAGGCCCCTGGCCCTGAACACTGTGGAGATGCTGCGTGTGGCCAGCTCTTCTCTGGGCATGGGGCCGCAGCACGCCATGCAGACGGCTGAGCGGCTCTACACGCAAGGCTACATCAGCTACCCAtggACAGAGACCACCCACTACCCTGAGAACTTTGACCTGAAGGGCTCTCTGCGGCAGCAGGCCAACCACCCCTACTGGGCCGACACGGTGAAGCGGTTGTTAGCAGAAGGTATCAACCGCCCGCGGAAAGGCCATGACGCCGGCGACCATCCCCCCATCACCCCCATGAAGTCTGCCACAGAGGCCGAATTAGGGGGTGACGCGTGGCGGCTCTATGAGTACATCACCAGACACTTCATCGCCACGGTCAGCCATGACTGCAAGTACCTGCAGAGCACCATCTCCTTCAGAATTGGGCCCGAGCTCTTCACCTGCTCCGGGAAGACCGTCCTCTCACCAGGCTTCACGGAGGTCATGCCCTGGCAGAGCGTGCCCCTGGAGGAGAGCCTGCCCACTTGCCAGCGGGGTGATGCCTTCCCTGTGGGCGAGGTGAAGATGCTGGAGAAGCAGACGAACCCACCCGACTACCTGACGGAGGCCGAGCTCATCACGCTCATGGAGAAGCATGGCATCGGCACGGATGCCAGCATCCCTGTGCATATCAACAACATCTGCCAGCGCAACTATGTCACGGTGGAGAGCGGGCGCCGGCTCAAGCCCACCAACCTCGGCATCGTCCTGGTGCACGGCTACTATAAGATTGATGCAGAGCTGGTGCTCCCCACCATCCGCAGTGCAGTGGAGAAGCAGCTGAACCTGATCGCCCAGGGCAAGGCCGACTACCGCCAGGTCCTGGGCCACACCCTGGACGTGTTCAAGAGGAAGTTCCACTACTTTGTCGACTCCATTGCTGGCATGGATGAGTTGATGGAGGTGTCTTTCTCGCCCCTGGCGGCCACAGGCAAGCCCCTCTCACGCTGTGGGAAGTGCCACCGCTTCATGAAGTACATCCAGGCCAAGCCAAGCCGCCTGCACTGCTCCCACTGCGATGAGACCTACACGCTCCCCCAGAACGGCACCATCAAGCTCTACAAGGAGCTCCGCTGCCCTCTGGATGACTTCGAGCTGGTCCTGTGGTCATCAGGCTCTCGGGGCAAGAGCTACCCGCTGTGCCCCTACTGCTACAACCACCCACCCTTCCGAGACATGAAGAAAGGCATGGGCTGCAACGAGTGTACGCACCCCTCCTGCCAGCACTCGCTGAGCATGCTGGGCATCGGCCAGTGCGTGGAATGTGAGAGCGGGGTGCTGGTGCTGGACCCCACCTCGGGCCCCAAGTGGAAGGTGGCCTGCAACAAGTGCAACGTGGTAGCGCACTGCTTCGAGAACGCCCACCGCGTGCGGGTGTCCGCCGACACCTGCAGTGTCTGTGAGGCCGCCTTGCTTGATGTGGACTTCAACAAGGCCAAGTCCCCACTCCCGGGCGATGAGACGCAGCACATGGGCTGCGTCTTTTGTGACCCCGTCTTCCAGGAGCTGGTGGAGCTGAAGCATGCGGCCTCCTGCCACCCCATGCACCGCGGTGGACCAGGGAGAAGGCAGGGTCGAGGGCGGGGCCGGGCCAGGAGGCCCCCTGGGAAGCCCAACCCCAGACGGCCCAAGGACAAGATGTCAGCCCTGGCCGCCTACTTTGTATAA

**3xFLAG-mEGFP-NES-TOP3B C666R (used in pGW1 and pcDNA5/FRT/TO)**

atgggaagcagcgactacaaagaccatgacggtgattataaagatcatgacatcgattacaaggatgacgatgacaagggttctggtgtgagcaagggcgaggagctgttcaccggggtggtgcccatcctggtcgagctggacggcgacgtaaacggccacaagttcagcgtgtccggcgagggcgagggcgatgccacctacggcaagctgaccctgaagttcatctgcaccaccggcaagctgcccgtgccctggcccaccctcgtgaccaccctgacctacggcgtgcagtgcttcagccgctaccccgaccacatgaagcagcacgacttcttcaagtccgccatgcccgaaggctacgtccaggagcgcaccatcttcttcaaggacgacggcaactacaagacccgcgccgaggtgaagttcgagggcgacaccctggtgaaccgcatcgagctgaagggcatcgacttcaaggaggacggcaacatcctggggcacaagctggagtacaactacaacagccacaacgtctatatcatggccgacaagcagaagaacggcatcaaggtgaacttcaagatccgccacaacatcgaggacggcagcgtgcagctcgccgaccactaccagcagaacacccccatcggcgacggccccgtgctgctgcccgacaaccactacctgagcacccagtccaagctgagcaaagaccccaacgagaagcgcgatcacatggtcctgctggagttcgtgaccgccgccgggatcactctcggcatggacgagctgtacaagggttctggtctgcagctgcctcccctggagcgcctgaccctggacggttcttctAAGACTGTGCTCATGGTTGCTGAAAAGCCGTCCTTGGCACAGTCAATTGCCAAAATCCTCTCTAGAGGGAGCCTGTCCTCACACAAAGGGCTGAACGGGGCCTGCTCAGTCCACGAGTACACTGGGACCTTTGCTGGCCAGCCAGTGCGCTTCAAGATGACGTCTGTCTGTGGTCACGTGATGACCCTGGATTTCCTGGGAAAATACAACAAATGGGACAAAGTGGACCCCGCAGAACTGTTCAGCCAAGCTCCCACGGAGAAGAAAGAAGCTAACCCCAAGCTGAACATGGTGAAGTTCCTGCAGGTGGAGGGCAGAGGCTGCGACTACATCGTGCTGTGGCTGGACTGCGACAAGGAGGGGGAGAACATCTGCTTTGAGGTTCTTGATGCTGTTCTGCCCGTCATGAACAAGGCCCATGGTGGCGAGAAGACCGTGTTCCGGGCCAGGTTTAGCTCCATCACGGACACAGACATCTGTAATGCCATGGCCTGCCTAGGCGAGCCTGACCACAACGAGGCGCTCTCAGTGGATGCTCGCCAGGAGCTGGACCTGCGAATCGGCTGTGCATTCACCAGGTTTCAGACTAAATATTTCCAGGGGAAATACGGTGATTTAGACAGCTCTCTCATCTCCTTTGGGCCGTGTCAGACTCCAACCCTGGGATTCTGTGTGGAGAGACATGATAAAATCCAGTCCTTCAAACCAGAGACCTACTGGGTGCTGCAGGCCAAGGTTAACACTGACAAAGACAGATCTCTCCTTTTGGACTGGGACCGAGTAAGAGTGTTTGACCGGGAGATCGCACAGATGTTTTTAAACATGACAAAGCTGGAGAAGGAAGCCCAGGTGGAGGCCACAAGCAGGAAAGAAAAGGCCAAGCAGAGGCCCCTGGCCCTGAACACTGTGGAGATGCTGCGTGTGGCCAGCTCTTCTCTGGGCATGGGGCCGCAGCACGCCATGCAGACGGCTGAGCGGCTCTACACGCAAGGCTACATCAGCTACCCACGGACAGAGACCACCCACTACCCTGAGAACTTTGACCTGAAGGGCTCTCTGCGGCAGCAGGCCAACCACCCCTACTGGGCCGACACGGTGAAGCGGTTGTTAGCAGAAGGTATCAACCGCCCGCGGAAAGGCCATGACGCCGGCGACCATCCCCCCATCACCCCCATGAAGTCTGCCACAGAGGCCGAATTAGGGGGTGACGCGTGGCGGCTCTATGAGTACATCACCAGACACTTCATCGCCACGGTCAGCCATGACTGCAAGTACCTGCAGAGCACCATCTCCTTCAGAATTGGGCCCGAGCTCTTCACCTGCTCCGGGAAGACCGTCCTCTCACCAGGCTTCACGGAGGTCATGCCCTGGCAGAGCGTGCCCCTGGAGGAGAGCCTGCCCACTTGCCAGCGGGGTGATGCCTTCCCTGTGGGCGAGGTGAAGATGCTGGAGAAGCAGACGAACCCACCCGACTACCTGACGGAGGCCGAGCTCATCACGCTCATGGAGAAGCATGGCATCGGCACGGATGCCAGCATCCCTGTGCATATCAACAACATCTGCCAGCGCAACTATGTCACGGTGGAGAGCGGGCGCCGGCTCAAGCCCACCAACCTCGGCATCGTCCTGGTGCACGGCTACTATAAGATTGATGCAGAGCTGGTGCTCCCCACCATCCGCAGTGCAGTGGAGAAGCAGCTGAACCTGATCGCCCAGGGCAAGGCCGACTACCGCCAGGTCCTGGGCCACACCCTGGACGTGTTCAAGAGGAAGTTCCACTACTTTGTCGACTCCATTGCTGGCATGGATGAGTTGATGGAGGTGTCTTTCTCGCCCCTGGCGGCCACAGGCAAGCCCCTCTCACGCTGTGGGAAGTGCCACCGCTTCATGAAGTACATCCAGGCCAAGCCAAGCCGCCTGCACTGCTCCCACTGCGATGAGACCTACACGCTCCCCCAGAACGGCACCATCAAGCTCTACAAGGAGCTCCGCcgcCCTCTGGATGACTTCGAGCTGGTCCTGTGGTCATCAGGCTCTCGGGGCAAGAGCTACCCGCTGTGCCCCTACTGCTACAACCACCCACCCTTCCGAGACATGAAGAAAGGCATGGGCTGCAACGAGTGTACGCACCCCTCCTGCCAGCACTCGCTGAGCATGCTGGGCATCGGCCAGTGCGTGGAATGTGAGAGCGGGGTGCTGGTGCTGGACCCCACCTCGGGCCCCAAGTGGAAGGTGGCCTGCAACAAGTGCAACGTGGTAGCGCACTGCTTCGAGAACGCCCACCGCGTGCGGGTGTCCGCCGACACCTGCAGTGTCTGTGAGGCCGCCTTGCTTGATGTGGACTTCAACAAGGCCAAGTCCCCACTCCCGGGCGATGAGACGCAGCACATGGGCTGCGTCTTTTGTGACCCCGTCTTCCAGGAGCTGGTGGAGCTGAAGCATGCGGCCTCCTGCCACCCCATGCACCGCGGTGGACCAGGGAGAAGGCAGGGTCGAGGGCGGGGCCGGGCCAGGAGGCCCCCTGGGAAGCCCAACCCCAGACGGCCCAAGGACAAGATGTCAGCCCTGGCCGCCTACTTTGTATAA

**ORFs in plasmids for recombinant protein expression and purification**

**In the plasmids below, mEGFP is under regulation of a separate promoter in the opposite direction than the MBP fusion.*

**mEGFP___FLAG-MBP-twinSTII**

TTACTTGTACAGCTCGTCCATGCCGAGAGTGATCCCGGCGGCGGTCACGAACTCCAGCAGGACCATGTGATCGCGCTTCTCGTTGGGGTCTTTGCTCAGCTTGGACTGGGTGCTCAGGTAGTGGTTGTCGGGCAGCAGCACGGGGCCGTCGCCGATGGGGGTGTTCTGCTGGTAGTGGTCGGCGAGCTGCACGCTGCCGTCCTCGATGTTGTGGCGGATCTTGAAGTTCACCTTGATGCCGTTCTTCTGCTTGTCGGCCATGATATAGACGTTGTGGCTGTTGTAGTTGTACTCCAGCTTGTGCCCCAGGATGTTGCCGTCCTCCTTGAAGTCGATGCCCTTCAGCTCGATGCGGTTCACCAGGGTGTCGCCCTCGAACTTCACCTCGGCGCGGGTCTTGTAGTTGCCGTCGTCCTTGAAGAAGATGGTGCGCTCCTGGACGTAGCCTTCGGGCATGGCGGACTTGAAGAAGTCGTGCTGCTTCATGTGGTCGGGGTAGCGGCTGAAGCACTGCACGCCGTAGGTCAGGGTGGTCACGAGGGTGGGCCAGGGCACGGGCAGCTTGCCGGTGGTGCAGATGAACTTCAGGGTCAGCTTGCCGTAGGTGGCATCGCCCTCGCCCTCGCCGGACACGCTGAACTTGTGGCCGTTTACGTCGCCGTCCAGCTCGACCAGGATGGGCACCACCCCGGTGAACAGCTCCTCGCCCTTGCTCACCATggtgGGTACCGCATGCTATGCATCAGCTGCTAGCACCATGGCTCGAGATCCCGGGTGATCAAGTCTTCGTCGAGTGATTGTAAATAAAATGTAATTTACAGTATAGTATTTTAATTAATATACAAATGATTTGATAATAATTCTTATTTAACTATAATATATTGTGTTGGGTTGAATTAAAGGTCCGTATACTCCGGAATATTAATAGATCATGGAGATAATTAAAATGATAACCATCTCGCAAATAAATAAGTATTTTACTGTTTTCGTAACAGTTTTGTAATAAAAAAACCTATAAATATTCCGGATTATTCATACCGTCCCACCATCGGGCGCGGATCCCGGTCCGAAGCGCGCGGAATTCcaccatgggcagcggcGATTACAAGGATGACGACGATAAGggttcttctatgaaaatcgaagaaggtaaactggtaatctggattaacggcgataaaggctataacggtctcgctgaagtcggtaagaaattcgagaaagataccggaattaaagtcaccgttgagcatccggataaactggaagagaaattcccacaggttgcggcaactggcgatggccctgacattatcttctgggcacacgaccgctttggtggctacgctcaatctggcctgttggctgaaatcaccccggacaaagcgttccaggacaagctgtatccgtttacctgggatgccgtacgttacaacggcaagctgattgcttacccgatcgctgttgaagcgttatcgctgatttataacaaagatctgctgccgaacccgccaaaaacctgggaagagatcccggcgctggataaagaactgaaagcgaaaggtaagagcgcgctgatgttcaacctgcaagaaccgtacttcacctggccgctgattgctgctgacgggggttatgcgttcaagtatgaaaacggcaagtacgacattaaagacgtgggcgtggataacgctggcgcgaaagcgggtctgaccttcctggttgacctgattaaaaacaaacacatgaatgcagacaccgattactccatcgcagaagctgcctttaataaaggcgaaacagcgatgaccatcaacggcccgtgggcatggtccaacatcgacaccagcaaagtgaattatggtgtaacggtactgccgaccttcaagggtcaaccatccaaaccgttcgttggcgtgctgagcgcaggtattaacgccgccagtccgaacaaagagctggcaaaagagttcctcgaaaactatctgctgactgatgaaggtctggaagcggttaataaagacaaaccgctgggtgccgtagcgctgaagtcttacgaggaagagttggcgaaagatccacgtattgccgccactatggaaaacgcccagaaaggtgaaatcatgccgaacatcccgcagatgtccgctttctggtatgccgtgcgtactgcggtgatcaacgccgccagcggtcgtcagactgtcgatgaagccctgaaagacgcgcagactaatgggatcgaggaaaacctgtacttccaatccggcagcggctggagccatccgcagtttgaaaaaggcagctggagccatccgcagtttgaaaaataa

**mEGFP___FLAG-MBP-ZnF (WT)-twinSTII**

TTACTTGTACAGCTCGTCCATGCCGAGAGTGATCCCGGCGGCGGTCACGAACTCCAGCAGGACCATGTGATCGCGCTTCTCGTTGGGGTCTTTGCTCAGCTTGGACTGGGTGCTCAGGTAGTGGTTGTCGGGCAGCAGCACGGGGCCGTCGCCGATGGGGGTGTTCTGCTGGTAGTGGTCGGCGAGCTGCACGCTGCCGTCCTCGATGTTGTGGCGGATCTTGAAGTTCACCTTGATGCCGTTCTTCTGCTTGTCGGCCATGATATAGACGTTGTGGCTGTTGTAGTTGTACTCCAGCTTGTGCCCCAGGATGTTGCCGTCCTCCTTGAAGTCGATGCCCTTCAGCTCGATGCGGTTCACCAGGGTGTCGCCCTCGAACTTCACCTCGGCGCGGGTCTTGTAGTTGCCGTCGTCCTTGAAGAAGATGGTGCGCTCCTGGACGTAGCCTTCGGGCATGGCGGACTTGAAGAAGTCGTGCTGCTTCATGTGGTCGGGGTAGCGGCTGAAGCACTGCACGCCGTAGGTCAGGGTGGTCACGAGGGTGGGCCAGGGCACGGGCAGCTTGCCGGTGGTGCAGATGAACTTCAGGGTCAGCTTGCCGTAGGTGGCATCGCCCTCGCCCTCGCCGGACACGCTGAACTTGTGGCCGTTTACGTCGCCGTCCAGCTCGACCAGGATGGGCACCACCCCGGTGAACAGCTCCTCGCCCTTGCTCACCATggtgGGTACCGCATGCTATGCATCAGCTGCTAGCACCATGGCTCGAGATCCCGGGTGATCAAGTCTTCGTCGAGTGATTGTAAATAAAATGTAATTTACAGTATAGTATTTTAATTAATATACAAATGATTTGATAATAATTCTTATTTAACTATAATATATTGTGTTGGGTTGAATTAAAGGTCCGTATACTCCGGAATATTAATAGATCATGGAGATAATTAAAATGATAACCATCTCGCAAATAAATAAGTATTTTACTGTTTTCGTAACAGTTTTGTAATAAAAAAACCTATAAATATTCCGGATTATTCATACCGTCCCACCATCGGGCGCGGATCCCGGTCCGAAGCGCGCGGAATTCcaccatgggcagcggcGATTACAAGGATGACGACGATAAGggttcttctatgaaaatcgaagaaggtaaactggtaatctggattaacggcgataaaggctataacggtctcgctgaagtcggtaagaaattcgagaaagataccggaattaaagtcaccgttgagcatccggataaactggaagagaaattcccacaggttgcggcaactggcgatggccctgacattatcttctgggcacacgaccgctttggtggctacgctcaatctggcctgttggctgaaatcaccccggacaaagcgttccaggacaagctgtatccgtttacctgggatgccgtacgttacaacggcaagctgattgcttacccgatcgctgttgaagcgttatcgctgatttataacaaagatctgctgccgaacccgccaaaaacctgggaagagatcccggcgctggataaagaactgaaagcgaaaggtaagagcgcgctgatgttcaacctgcaagaaccgtacttcacctggccgctgattgctgctgacgggggttatgcgttcaagtatgaaaacggcaagtacgacattaaagacgtgggcgtggataacgctggcgcgaaagcgggtctgaccttcctggttgacctgattaaaaacaaacacatgaatgcagacaccgattactccatcgcagaagctgcctttaataaaggcgaaacagcgatgaccatcaacggcccgtgggcatggtccaacatcgacaccagcaaagtgaattatggtgtaacggtactgccgaccttcaagggtcaaccatccaaaccgttcgttggcgtgctgagcgcaggtattaacgccgccagtccgaacaaagagctggcaaaagagttcctcgaaaactatctgctgactgatgaaggtctggaagcggttaataaagacaaaccgctgggtgccgtagcgctgaagtcttacgaggaagagttggcgaaagatccacgtattgccgccactatggaaaacgcccagaaaggtgaaatcatgccgaacatcccgcagatgtccgctttctggtatgccgtgcgtactgcggtgatcaacgccgccagcggtcgtcagactgtcgatgaagccctgaaagacgcgcagactaatgggatcgaggaaaacctgtacttccaatccaatattggaagtggaggcaagcccctctcacgctgtgggaagtgccaccgcttcatgaagtacatccaggccaagccaagccgcctgcactgctcccactgcgatgagacctacacgctcccccagaacggcaccatcaagctctacaaggagctccgctgccctctggatgacttcgagctggtcctgtggtcatcaggctctcggggcaagagctacccgctgtgcccctactgctacaaccacccacccttccgagacatgaagaaaggcatgggctgcaacgagtgtacgcacccctcctgccagcactcgctgagcatgctgggcatcggccagtgcgtggaatgtgagagcggggtgctggtgctggaccccacctcgggccccaagtggaaggtggcctgcaacaagtgcaacgtggtagcgcactgcttcgagaacgcccaccgcgtgcgggtgtccgccgacacctgcagtgtctgtgaggccgccttgcttgatgtggacttcaacaaggccaagtccccactcccgggcgatgagacgcagcacatgggctgcgtcttttgtgaccccgtcttccaggagctggtggagctgaagcatgcggcctccggcagcggctggagccatccgcagtttgaaaaaggcagctggagccatccgcagtttgaaaaataa

**mEGFP___FLAG-MBP-ZnF (C666R)-twinSTII**

TTACTTGTACAGCTCGTCCATGCCGAGAGTGATCCCGGCGGCGGTCACGAACTCCAGCAGGACCATGTGATCGCGCTTCTCGTTGGGGTCTTTGCTCAGCTTGGACTGGGTGCTCAGGTAGTGGTTGTCGGGCAGCAGCACGGGGCCGTCGCCGATGGGGGTGTTCTGCTGGTAGTGGTCGGCGAGCTGCACGCTGCCGTCCTCGATGTTGTGGCGGATCTTGAAGTTCACCTTGATGCCGTTCTTCTGCTTGTCGGCCATGATATAGACGTTGTGGCTGTTGTAGTTGTACTCCAGCTTGTGCCCCAGGATGTTGCCGTCCTCCTTGAAGTCGATGCCCTTCAGCTCGATGCGGTTCACCAGGGTGTCGCCCTCGAACTTCACCTCGGCGCGGGTCTTGTAGTTGCCGTCGTCCTTGAAGAAGATGGTGCGCTCCTGGACGTAGCCTTCGGGCATGGCGGACTTGAAGAAGTCGTGCTGCTTCATGTGGTCGGGGTAGCGGCTGAAGCACTGCACGCCGTAGGTCAGGGTGGTCACGAGGGTGGGCCAGGGCACGGGCAGCTTGCCGGTGGTGCAGATGAACTTCAGGGTCAGCTTGCCGTAGGTGGCATCGCCCTCGCCCTCGCCGGACACGCTGAACTTGTGGCCGTTTACGTCGCCGTCCAGCTCGACCAGGATGGGCACCACCCCGGTGAACAGCTCCTCGCCCTTGCTCACCATggtgGGTACCGCATGCTATGCATCAGCTGCTAGCACCATGGCTCGAGATCCCGGGTGATCAAGTCTTCGTCGAGTGATTGTAAATAAAATGTAATTTACAGTATAGTATTTTAATTAATATACAAATGATTTGATAATAATTCTTATTTAACTATAATATATTGTGTTGGGTTGAATTAAAGGTCCGTATACTCCGGAATATTAATAGATCATGGAGATAATTAAAATGATAACCATCTCGCAAATAAATAAGTATTTTACTGTTTTCGTAACAGTTTTGTAATAAAAAAACCTATAAATATTCCGGATTATTCATACCGTCCCACCATCGGGCGCGGATCCCGGTCCGAAGCGCGCGGAATTCcaccatgggcagcggcGATTACAAGGATGACGACGATAAGggttcttctatgaaaatcgaagaaggtaaactggtaatctggattaacggcgataaaggctataacggtctcgctgaagtcggtaagaaattcgagaaagataccggaattaaagtcaccgttgagcatccggataaactggaagagaaattcccacaggttgcggcaactggcgatggccctgacattatcttctgggcacacgaccgctttggtggctacgctcaatctggcctgttggctgaaatcaccccggacaaagcgttccaggacaagctgtatccgtttacctgggatgccgtacgttacaacggcaagctgattgcttacccgatcgctgttgaagcgttatcgctgatttataacaaagatctgctgccgaacccgccaaaaacctgggaagagatcccggcgctggataaagaactgaaagcgaaaggtaagagcgcgctgatgttcaacctgcaagaaccgtacttcacctggccgctgattgctgctgacgggggttatgcgttcaagtatgaaaacggcaagtacgacattaaagacgtgggcgtggataacgctggcgcgaaagcgggtctgaccttcctggttgacctgattaaaaacaaacacatgaatgcagacaccgattactccatcgcagaagctgcctttaataaaggcgaaacagcgatgaccatcaacggcccgtgggcatggtccaacatcgacaccagcaaagtgaattatggtgtaacggtactgccgaccttcaagggtcaaccatccaaaccgttcgttggcgtgctgagcgcaggtattaacgccgccagtccgaacaaagagctggcaaaagagttcctcgaaaactatctgctgactgatgaaggtctggaagcggttaataaagacaaaccgctgggtgccgtagcgctgaagtcttacgaggaagagttggcgaaagatccacgtattgccgccactatggaaaacgcccagaaaggtgaaatcatgccgaacatcccgcagatgtccgctttctggtatgccgtgcgtactgcggtgatcaacgccgccagcggtcgtcagactgtcgatgaagccctgaaagacgcgcagactaatgggatcgaggaaaacctgtacttccaatccaatattggaagtggaggcaagcccctctcacgctgtgggaagtgccaccgcttcatgaagtacatccaggccaagccaagccgcctgcactgctcccactgcgatgagacctacacgctcccccagaacggcaccatcaagctctacaaggagctccgccgccctctggatgacttcgagctggtcctgtggtcatcaggctctcggggcaagagctacccgctgtgcccctactgctacaaccacccacccttccgagacatgaagaaaggcatgggctgcaacgagtgtacgcacccctcctgccagcactcgctgagcatgctgggcatcggccagtgcgtggaatgtgagagcggggtgctggtgctggaccccacctcgggccccaagtggaaggtggcctgcaacaagtgcaacgtggtagcgcactgcttcgagaacgcccaccgcgtgcgggtgtccgccgacacctgcagtgtctgtgaggccgccttgcttgatgtggacttcaacaaggccaagtccccactcccgggcgatgagacgcagcacatgggctgcgtcttttgtgaccccgtcttccaggagctggtggagctgaagcatgcggcctccggcagcggctggagccatccgcagtttgaaaaaggcagctggagccatccgcagtttgaaaaataa

**mEGFP___FLAG-MBP-ZnF (D669A)-twinSTII**

TTACTTGTACAGCTCGTCCATGCCGAGAGTGATCCCGGCGGCGGTCACGAACTCCAGCAGGACCATGTGATCGCGCTTCTCGTTGGGGTCTTTGCTCAGCTTGGACTGGGTGCTCAGGTAGTGGTTGTCGGGCAGCAGCACGGGGCCGTCGCCGATGGGGGTGTTCTGCTGGTAGTGGTCGGCGAGCTGCACGCTGCCGTCCTCGATGTTGTGGCGGATCTTGAAGTTCACCTTGATGCCGTTCTTCTGCTTGTCGGCCATGATATAGACGTTGTGGCTGTTGTAGTTGTACTCCAGCTTGTGCCCCAGGATGTTGCCGTCCTCCTTGAAGTCGATGCCCTTCAGCTCGATGCGGTTCACCAGGGTGTCGCCCTCGAACTTCACCTCGGCGCGGGTCTTGTAGTTGCCGTCGTCCTTGAAGAAGATGGTGCGCTCCTGGACGTAGCCTTCGGGCATGGCGGACTTGAAGAAGTCGTGCTGCTTCATGTGGTCGGGGTAGCGGCTGAAGCACTGCACGCCGTAGGTCAGGGTGGTCACGAGGGTGGGCCAGGGCACGGGCAGCTTGCCGGTGGTGCAGATGAACTTCAGGGTCAGCTTGCCGTAGGTGGCATCGCCCTCGCCCTCGCCGGACACGCTGAACTTGTGGCCGTTTACGTCGCCGTCCAGCTCGACCAGGATGGGCACCACCCCGGTGAACAGCTCCTCGCCCTTGCTCACCATggtgGGTACCGCATGCTATGCATCAGCTGCTAGCACCATGGCTCGAGATCCCGGGTGATCAAGTCTTCGTCGAGTGATTGTAAATAAAATGTAATTTACAGTATAGTATTTTAATTAATATACAAATGATTTGATAATAATTCTTATTTAACTATAATATATTGTGTTGGGTTGAATTAAAGGTCCGTATACTCCGGAATATTAATAGATCATGGAGATAATTAAAATGATAACCATCTCGCAAATAAATAAGTATTTTACTGTTTTCGTAACAGTTTTGTAATAAAAAAACCTATAAATATTCCGGATTATTCATACCGTCCCACCATCGGGCGCGGATCCCGGTCCGAAGCGCGCGGAATTCcaccatgggcagcggcGATTACAAGGATGACGACGATAAGggttcttctatgaaaatcgaagaaggtaaactggtaatctggattaacggcgataaaggctataacggtctcgctgaagtcggtaagaaattcgagaaagataccggaattaaagtcaccgttgagcatccggataaactggaagagaaattcccacaggttgcggcaactggcgatggccctgacattatcttctgggcacacgaccgctttggtggctacgctcaatctggcctgttggctgaaatcaccccggacaaagcgttccaggacaagctgtatccgtttacctgggatgccgtacgttacaacggcaagctgattgcttacccgatcgctgttgaagcgttatcgctgatttataacaaagatctgctgccgaacccgccaaaaacctgggaagagatcccggcgctggataaagaactgaaagcgaaaggtaagagcgcgctgatgttcaacctgcaagaaccgtacttcacctggccgctgattgctgctgacgggggttatgcgttcaagtatgaaaacggcaagtacgacattaaagacgtgggcgtggataacgctggcgcgaaagcgggtctgaccttcctggttgacctgattaaaaacaaacacatgaatgcagacaccgattactccatcgcagaagctgcctttaataaaggcgaaacagcgatgaccatcaacggcccgtgggcatggtccaacatcgacaccagcaaagtgaattatggtgtaacggtactgccgaccttcaagggtcaaccatccaaaccgttcgttggcgtgctgagcgcaggtattaacgccgccagtccgaacaaagagctggcaaaagagttcctcgaaaactatctgctgactgatgaaggtctggaagcggttaataaagacaaaccgctgggtgccgtagcgctgaagtcttacgaggaagagttggcgaaagatccacgtattgccgccactatggaaaacgcccagaaaggtgaaatcatgccgaacatcccgcagatgtccgctttctggtatgccgtgcgtactgcggtgatcaacgccgccagcggtcgtcagactgtcgatgaagccctgaaagacgcgcagactaatgggatcgaggaaaacctgtacttccaatccaatattggaagtggaggcaagcccctctcacgctgtgggaagtgccaccgcttcatgaagtacatccaggccaagccaagccgcctgcactgctcccactgcgatgagacctacacgctcccccagaacggcaccatcaagctctacaaggagctccgctgccctctggccgacttcgagctggtcctgtggtcatcaggctctcggggcaagagctacccgctgtgcccctactgctacaaccacccacccttccgagacatgaagaaaggcatgggctgcaacgagtgtacgcacccctcctgccagcactcgctgagcatgctgggcatcggccagtgcgtggaatgtgagagcggggtgctggtgctggaccccacctcgggccccaagtggaaggtggcctgcaacaagtgcaacgtggtagcgcactgcttcgagaacgcccaccgcgtgcgggtgtccgccgacacctgcagtgtctgtgaggccgccttgcttgatgtggacttcaacaaggccaagtccccactcccgggcgatgagacgcagcacatgggctgcgtcttttgtgaccccgtcttccaggagctggtggagctgaagcatgcggcctccggcagcggctggagccatccgcagtttgaaaaaggcagctggagccatccgcagtttgaaaaataa

**mEGFP___FLAG-MBP-ZnF (C688A/C691A)-twinSTII**

TTACTTGTACAGCTCGTCCATGCCGAGAGTGATCCCGGCGGCGGTCACGAACTCCAGCAGGACCATGTGATCGCGCTTCTCGTTGGGGTCTTTGCTCAGCTTGGACTGGGTGCTCAGGTAGTGGTTGTCGGGCAGCAGCACGGGGCCGTCGCCGATGGGGGTGTTCTGCTGGTAGTGGTCGGCGAGCTGCACGCTGCCGTCCTCGATGTTGTGGCGGATCTTGAAGTTCACCTTGATGCCGTTCTTCTGCTTGTCGGCCATGATATAGACGTTGTGGCTGTTGTAGTTGTACTCCAGCTTGTGCCCCAGGATGTTGCCGTCCTCCTTGAAGTCGATGCCCTTCAGCTCGATGCGGTTCACCAGGGTGTCGCCCTCGAACTTCACCTCGGCGCGGGTCTTGTAGTTGCCGTCGTCCTTGAAGAAGATGGTGCGCTCCTGGACGTAGCCTTCGGGCATGGCGGACTTGAAGAAGTCGTGCTGCTTCATGTGGTCGGGGTAGCGGCTGAAGCACTGCACGCCGTAGGTCAGGGTGGTCACGAGGGTGGGCCAGGGCACGGGCAGCTTGCCGGTGGTGCAGATGAACTTCAGGGTCAGCTTGCCGTAGGTGGCATCGCCCTCGCCCTCGCCGGACACGCTGAACTTGTGGCCGTTTACGTCGCCGTCCAGCTCGACCAGGATGGGCACCACCCCGGTGAACAGCTCCTCGCCCTTGCTCACCATggtgGGTACCGCATGCTATGCATCAGCTGCTAGCACCATGGCTCGAGATCCCGGGTGATCAAGTCTTCGTCGAGTGATTGTAAATAAAATGTAATTTACAGTATAGTATTTTAATTAATATACAAATGATTTGATAATAATTCTTATTTAACTATAATATATTGTGTTGGGTTGAATTAAAGGTCCGTATACTCCGGAATATTAATAGATCATGGAGATAATTAAAATGATAACCATCTCGCAAATAAATAAGTATTTTACTGTTTTCGTAACAGTTTTGTAATAAAAAAACCTATAAATATTCCGGATTATTCATACCGTCCCACCATCGGGCGCGGATCCCGGTCCGAAGCGCGCGGAATTCcaccatgggcagcggcGATTACAAGGATGACGACGATAAGggttcttctatgaaaatcgaagaaggtaaactggtaatctggattaacggcgataaaggctataacggtctcgctgaagtcggtaagaaattcgagaaagataccggaattaaagtcaccgttgagcatccggataaactggaagagaaattcccacaggttgcggcaactggcgatggccctgacattatcttctgggcacacgaccgctttggtggctacgctcaatctggcctgttggctgaaatcaccccggacaaagcgttccaggacaagctgtatccgtttacctgggatgccgtacgttacaacggcaagctgattgcttacccgatcgctgttgaagcgttatcgctgatttataacaaagatctgctgccgaacccgccaaaaacctgggaagagatcccggcgctggataaagaactgaaagcgaaaggtaagagcgcgctgatgttcaacctgcaagaaccgtacttcacctggccgctgattgctgctgacgggggttatgcgttcaagtatgaaaacggcaagtacgacattaaagacgtgggcgtggataacgctggcgcgaaagcgggtctgaccttcctggttgacctgattaaaaacaaacacatgaatgcagacaccgattactccatcgcagaagctgcctttaataaaggcgaaacagcgatgaccatcaacggcccgtgggcatggtccaacatcgacaccagcaaagtgaattatggtgtaacggtactgccgaccttcaagggtcaaccatccaaaccgttcgttggcgtgctgagcgcaggtattaacgccgccagtccgaacaaagagctggcaaaagagttcctcgaaaactatctgctgactgatgaaggtctggaagcggttaataaagacaaaccgctgggtgccgtagcgctgaagtcttacgaggaagagttggcgaaagatccacgtattgccgccactatggaaaacgcccagaaaggtgaaatcatgccgaacatcccgcagatgtccgctttctggtatgccgtgcgtactgcggtgatcaacgccgccagcggtcgtcagactgtcgatgaagccctgaaagacgcgcagactaatgggatcgaggaaaacctgtacttccaatccaatattggaagtggaggcaagcccctctcacgctgtgggaagtgccaccgcttcatgaagtacatccaggccaagccaagccgcctgcactgctcccactgcgatgagacctacacgctcccccagaacggcaccatcaagctctacaaggagctccgctgccctctggatgacttcgagctggtcctgtggtcatcaggctctcggggcaagagctacccgctggccccctacgcctacaaccacccacccttccgagacatgaagaaaggcatgggctgcaacgagtgtacgcacccctcctgccagcactcgctgagcatgctgggcatcggccagtgcgtggaatgtgagagcggggtgctggtgctggaccccacctcgggccccaagtggaaggtggcctgcaacaagtgcaacgtggtagcgcactgcttcgagaacgcccaccgcgtgcgggtgtccgccgacacctgcagtgtctgtgaggccgccttgcttgatgtggacttcaacaaggccaagtccccactcccgggcgatgagacgcagcacatgggctgcgtcttttgtgaccccgtcttccaggagctggtggagctgaagcatgcggcctccggcagcggctggagccatccgcagtttgaaaaaggcagctggagccatccgcagtttgaaaaataa

**His6-TOP3B(WT)-FLAG**

ATGggttcttctcaccatcaccatcaccatggttcttctAAGACTGTGCTCATGGTTGCTGAAAAGCCGTCCTTGGCACAGTCAATTGCCAAAATCCTCTCTAGAGGGAGCCTGTCCTCACACAAAGGGCTGAACGGGGCCTGCTCAGTCCACGAGTACACTGGGACCTTTGCTGGCCAGCCAGTGCGCTTCAAGATGACGTCTGTCTGTGGTCACGTGATGACCCTGGATTTCCTGGGAAAATACAACAAATGGGACAAAGTGGACCCCGCAGAACTGTTCAGCCAAGCTCCCACGGAGAAGAAAGAAGCTAACCCCAAGCTGAACATGGTGAAGTTCCTGCAGGTGGAGGGCAGAGGCTGCGACTACATCGTGCTGTGGCTGGACTGCGACAAGGAGGGGGAGAACATCTGCTTTGAGGTTCTTGATGCTGTTCTGCCCGTCATGAACAAGGCCCATGGTGGCGAGAAGACCGTGTTCCGGGCCAGGTTTAGCTCCATCACGGACACAGACATCTGTAATGCCATGGCCTGCCTAGGCGAGCCTGACCACAACGAGGCGCTCTCAGTGGATGCTCGCCAGGAGCTGGACCTGCGAATCGGCTGTGCATTCACCAGGTTTCAGACTAAATATTTCCAGGGGAAATACGGTGATTTAGACAGCTCTCTCATCTCCTTTGGGCCGTGTCAGACTCCAACCCTGGGATTCTGTGTGGAGAGACATGATAAAATCCAGTCCTTCAAACCAGAGACCTACTGGGTGCTGCAGGCCAAGGTTAACACTGACAAAGACAGATCTCTCCTTTTGGACTGGGACCGAGTAAGAGTGTTTGACCGGGAGATCGCACAGATGTTTTTAAACATGACAAAGCTGGAGAAGGAAGCCCAGGTGGAGGCCACAAGCAGGAAAGAAAAGGCCAAGCAGAGGCCCCTGGCCCTGAACACTGTGGAGATGCTGCGTGTGGCCAGCTCTTCTCTGGGCATGGGGCCGCAGCACGCCATGCAGACGGCTGAGCGGCTCTACACGCAAGGCTACATCAGCTACCCACGGACAGAGACCACCCACTACCCTGAGAACTTTGACCTGAAGGGCTCTCTGCGGCAGCAGGCCAACCACCCCTACTGGGCCGACACGGTGAAGCGGTTGTTAGCAGAAGGTATCAACCGCCCGCGGAAAGGCCATGACGCCGGCGACCATCCCCCCATCACCCCCATGAAGTCTGCCACAGAGGCCGAATTAGGGGGTGACGCGTGGCGGCTCTATGAGTACATCACCAGACACTTCATCGCCACGGTCAGCCATGACTGCAAGTACCTGCAGAGCACCATCTCCTTCAGAATTGGGCCCGAGCTCTTCACCTGCTCCGGGAAGACCGTCCTCTCACCAGGCTTCACGGAGGTCATGCCCTGGCAGAGCGTGCCCCTGGAGGAGAGCCTGCCCACTTGCCAGCGGGGTGATGCCTTCCCTGTGGGCGAGGTGAAGATGCTGGAGAAGCAGACGAACCCACCCGACTACCTGACGGAGGCCGAGCTCATCACGCTCATGGAGAAGCATGGCATCGGCACGGATGCCAGCATCCCTGTGCATATCAACAACATCTGCCAGCGCAACTATGTCACGGTGGAGAGCGGGCGCCGGCTCAAGCCCACCAACCTCGGCATCGTCCTGGTGCACGGCTACTATAAGATTGATGCAGAGCTGGTGCTCCCCACCATCCGCAGTGCAGTGGAGAAGCAGCTGAACCTGATCGCCCAGGGCAAGGCCGACTACCGCCAGGTCCTGGGCCACACCCTGGACGTGTTCAAGAGGAAGTTCCACTACTTTGTCGACTCCATTGCTGGCATGGATGAGTTGATGGAGGTGTCTTTCTCGCCCCTGGCGGCCACAGGCAAGCCCCTCTCACGCTGTGGGAAGTGCCACCGCTTCATGAAGTACATCCAGGCCAAGCCAAGCCGCCTGCACTGCTCCCACTGCGATGAGACCTACACGCTCCCCCAGAACGGCACCATCAAGCTCTACAAGGAGCTCCGCTGCCCTCTGGATGACTTCGAGCTGGTCCTGTGGTCATCAGGCTCTCGGGGCAAGAGCTACCCGCTGTGCCCCTACTGCTACAACCACCCACCCTTCCGAGACATGAAGAAAGGCATGGGCTGCAACGAGTGTACGCACCCCTCCTGCCAGCACTCGCTGAGCATGCTGGGCATCGGCCAGTGCGTGGAATGTGAGAGCGGGGTGCTGGTGCTGGACCCCACCTCGGGCCCCAAGTGGAAGGTGGCCTGCAACAAGTGCAACGTGGTAGCGCACTGCTTCGAGAACGCCCACCGCGTGCGGGTGTCCGCCGACACCTGCAGTGTCTGTGAGGCCGCCTTGCTTGATGTGGACTTCAACAAGGCCAAGTCCCCACTCCCGGGCGATGAGACGCAGCACATGGGCTGCGTCTTTTGTGACCCCGTCTTCCAGGAGCTGGTGGAGCTGAAGCATGCGGCCTCCTGCCACCCCATGCACCGCGGTGGACCAGGGAGAAGGCAGGGTCGAGGGCGGGGCCGGGCCAGGAGGCCCCCTGGGAAGCCCAACCCCAGACGGCCCAAGGACAAGATGTCAGCCCTGGCCGCCTACTTTGTAAGCGGACCGACGCGTACGCGGCCGCTCGAGCAGAAACTCATCTCAGAAGAGGATCTGGCAGCAAATGATATCCTGGATTACAAGGATGACGACGATAAGGTTTAA

**His6-TOP3B(Y336F)-FLAG**

ATGggttcttctcaccatcaccatcaccatggttcttctAAGACTGTGCTCATGGTTGCTGAAAAGCCGTCCTTGGCACAGTCAATTGCCAAAATCCTCTCTAGAGGGAGCCTGTCCTCACACAAAGGGCTGAACGGGGCCTGCTCAGTCCACGAGTACACTGGGACCTTTGCTGGCCAGCCAGTGCGCTTCAAGATGACGTCTGTCTGTGGTCACGTGATGACCCTGGATTTCCTGGGAAAATACAACAAATGGGACAAAGTGGACCCCGCAGAACTGTTCAGCCAAGCTCCCACGGAGAAGAAAGAAGCTAACCCCAAGCTGAACATGGTGAAGTTCCTGCAGGTGGAGGGCAGAGGCTGCGACTACATCGTGCTGTGGCTGGACTGCGACAAGGAGGGGGAGAACATCTGCTTTGAGGTTCTTGATGCTGTTCTGCCCGTCATGAACAAGGCCCATGGTGGCGAGAAGACCGTGTTCCGGGCCAGGTTTAGCTCCATCACGGACACAGACATCTGTAATGCCATGGCCTGCCTAGGCGAGCCTGACCACAACGAGGCGCTCTCAGTGGATGCTCGCCAGGAGCTGGACCTGCGAATCGGCTGTGCATTCACCAGGTTTCAGACTAAATATTTCCAGGGGAAATACGGTGATTTAGACAGCTCTCTCATCTCCTTTGGGCCGTGTCAGACTCCAACCCTGGGATTCTGTGTGGAGAGACATGATAAAATCCAGTCCTTCAAACCAGAGACCTACTGGGTGCTGCAGGCCAAGGTTAACACTGACAAAGACAGATCTCTCCTTTTGGACTGGGACCGAGTAAGAGTGTTTGACCGGGAGATCGCACAGATGTTTTTAAACATGACAAAGCTGGAGAAGGAAGCCCAGGTGGAGGCCACAAGCAGGAAAGAAAAGGCCAAGCAGAGGCCCCTGGCCCTGAACACTGTGGAGATGCTGCGTGTGGCCAGCTCTTCTCTGGGCATGGGGCCGCAGCACGCCATGCAGACGGCTGAGCGGCTCTACACGCAAGGCTACATCAGCtttCCACGGACAGAGACCACCCACTACCCTGAGAACTTTGACCTGAAGGGCTCTCTGCGGCAGCAGGCCAACCACCCCTACTGGGCCGACACGGTGAAGCGGTTGTTAGCAGAAGGTATCAACCGCCCGCGGAAAGGCCATGACGCCGGCGACCATCCCCCCATCACCCCCATGAAGTCTGCCACAGAGGCCGAATTAGGGGGTGACGCGTGGCGGCTCTATGAGTACATCACCAGACACTTCATCGCCACGGTCAGCCATGACTGCAAGTACCTGCAGAGCACCATCTCCTTCAGAATTGGGCCCGAGCTCTTCACCTGCTCCGGGAAGACCGTCCTCTCACCAGGCTTCACGGAGGTCATGCCCTGGCAGAGCGTGCCCCTGGAGGAGAGCCTGCCCACTTGCCAGCGGGGTGATGCCTTCCCTGTGGGCGAGGTGAAGATGCTGGAGAAGCAGACGAACCCACCCGACTACCTGACGGAGGCCGAGCTCATCACGCTCATGGAGAAGCATGGCATCGGCACGGATGCCAGCATCCCTGTGCATATCAACAACATCTGCCAGCGCAACTATGTCACGGTGGAGAGCGGGCGCCGGCTCAAGCCCACCAACCTCGGCATCGTCCTGGTGCACGGCTACTATAAGATTGATGCAGAGCTGGTGCTCCCCACCATCCGCAGTGCAGTGGAGAAGCAGCTGAACCTGATCGCCCAGGGCAAGGCCGACTACCGCCAGGTCCTGGGCCACACCCTGGACGTGTTCAAGAGGAAGTTCCACTACTTTGTCGACTCCATTGCTGGCATGGATGAGTTGATGGAGGTGTCTTTCTCGCCCCTGGCGGCCACAGGCAAGCCCCTCTCACGCTGTGGGAAGTGCCACCGCTTCATGAAGTACATCCAGGCCAAGCCAAGCCGCCTGCACTGCTCCCACTGCGATGAGACCTACACGCTCCCCCAGAACGGCACCATCAAGCTCTACAAGGAGCTCCGCTGCCCTCTGGATGACTTCGAGCTGGTCCTGTGGTCATCAGGCTCTCGGGGCAAGAGCTACCCGCTGTGCCCCTACTGCTACAACCACCCACCCTTCCGAGACATGAAGAAAGGCATGGGCTGCAACGAGTGTACGCACCCCTCCTGCCAGCACTCGCTGAGCATGCTGGGCATCGGCCAGTGCGTGGAATGTGAGAGCGGGGTGCTGGTGCTGGACCCCACCTCGGGCCCCAAGTGGAAGGTGGCCTGCAACAAGTGCAACGTGGTAGCGCACTGCTTCGAGAACGCCCACCGCGTGCGGGTGTCCGCCGACACCTGCAGTGTCTGTGAGGCCGCCTTGCTTGATGTGGACTTCAACAAGGCCAAGTCCCCACTCCCGGGCGATGAGACGCAGCACATGGGCTGCGTCTTTTGTGACCCCGTCTTCCAGGAGCTGGTGGAGCTGAAGCATGCGGCCTCCTGCCACCCCATGCACCGCGGTGGACCAGGGAGAAGGCAGGGTCGAGGGCGGGGCCGGGCCAGGAGGCCCCCTGGGAAGCCCAACCCCAGACGGCCCAAGGACAAGATGTCAGCCCTGGCCGCCTACTTTGTAAGCGGACCGACGCGTACGCGGCCGCTCGAGCAGAAACTCATCTCAGAAGAGGATCTGGCAGCAAATGATATCCTGGATTACAAGGATGACGACGATAAGGTTTAA
